# Prenatal Reflective Functioning as a Predictor of Substance-Using Mothers' Treatment Outcome: Comparing Results From Two Different RF Measures

**DOI:** 10.3389/fpsyg.2022.909414

**Published:** 2022-07-25

**Authors:** Marjo Flykt, Ritva Belt, Saara Salo, Marjukka Pajulo, Raija-Leena Punamäki

**Affiliations:** ^1^Faculty of Medicine, Department of Psychology and Logopedics, University of Helsinki, Helsinki, Finland; ^2^Faculty of Social Sciences, Department of Psychology, Tampere University, Tampere, Finland; ^3^Department of Social Services, Tampere, Finland; ^4^Faculty of Education, University of Helsinki, Helsinki, Finland; ^5^Faculty of Medicine, Department of Child Psychiatry, University of Turku, Turku, Finland

**Keywords:** reflective functioning, mentalization, mother-infant interaction, substance use, intervention, attachment, parenting, prenatal

## Abstract

Mothers with prenatal substance use disorder (SUD) often show broad deficits in their reflective functioning (RF), implying severe risk for the relationship with their baby. Two different types of prenatal maternal RF may be important for parenting: adult attachment-focused-RF (AAI-RF), regarding parent's own childhood experiences, and parenting-focused RF (PRF) regarding their own current process of becoming a parent. However, their inter-relations and potentially different roles for parenting intervention outcomes are not clear. This study examined the associations between mothers' prenatal AAI-RF and pre- and post-natal PRF, and their role in mother-infant interaction and substance use as treatment outcomes. The participants were 57 treatment-enrolled pregnant mothers with SUD and 50 low-risk comparison mothers. AAI-RF was measured with the Adult Attachment Interview. For a subsample of 30 mothers with SUD, PRF was measured with Pregnancy Interview (during pregnancy/pre-intervention), and with Parent Development Interview at 4 months (during intervention). Mother-infant interaction was measured with Emotional Availability Scales at 4 and 12 months (post-intervention), and maternal substance use by post-natal substance relapses. Prenatal AAI-RF and pre- and post-natal PRF were highly associated with each other. Only higher prenatal PRF predicted better mother-infant interaction quality at 4 months and less substance use during the child's first year. Interestingly, prenatal PRF and AAI-RF predicted opposite changes in mother-infant interaction: lower prenatal PRF, but higher AAI-RF predicting more positive change. AAI-RF was especially associated with a change in maternal intrusiveness and hostility, indicating that it represents a more general regulatory tendency. Further studies are needed in larger and lower-risk samples. Our results suggest, however, that AAI-RF and PRF are partially distinct and should be uniquely targeted in perinatal interventions.

## Introduction

Parental reflective functioning (RF), the ability to understand behavior in terms of underlying mental states, such as feelings, desires, and intentions, is a fundamental characteristic of adaptive caregiving and contributes to the intergenerational transmission of attachment and trauma (Fonagy et al., 1991; Smaling et al., 2017). Most previous research has concerned post-natal RF, although prenatal RF is vital in revealing how maternal RF capacity acquired in her earlier attachment relationships affects the development of caregiving before the influence of child characteristics. Most studies on maternal RF and parenting have examined ***current parenting-focused RF*** (PRF), indicating maternal ability to reflect upon her child's and her own mental states. Higher PRF is known to associate with more optimal parent-infant interaction quality and child development, including child attachment security (See for a review, Camoirano, 2017). However, less is known about the role of maternal ability to reflect upon her own childhood attachment experiences, that is, her ***adult attachment-focused RF*** (AAI-RF), although it has also been associated with child attachment security and higher parent-infant interaction quality (Fonagy et al., 1991; Ensink et al., 2016). A recent review recommends examining the possible similarities and differences between AAI-RF and PRF (Camoirano, 2017), as their distinct contributions to intervention outcomes, such as parent-child interaction quality, are not yet understood.

A mother's prenatal substance use disorder (SUD) indicates a combination of severe, biopsychosocial risks for a healthy mother-child relationship and child development (Conners et al., 2004; Flykt et al., 2021). These include direct substance effects on the mother and the fetus, but typically also more distal risks, such as socioeconomic problems, mental health symptoms, and history of attachment insecurity and trauma. Mothers with SUD tend to show disturbed dyadic interaction patterns with their children, including insensitivity, hostility, intrusiveness, and withdrawal (Salo et al., 2009; Belt et al., 2012; Frigerio et al., 2019). Poor pre- and post-natal capacity for RF seems to underlie parenting difficulties in mothers with SUD, as well as the capacity to recover from addiction (Suchman et al., 2010; Pajulo et al., 2012), making RF an especially relevant target for perinatal substance use interventions. Mentalizing- and attachment-based interventions are known to enhance mother-infant interaction quality among mothers with SUD and their children and support abstinence from substances (Pajulo et al., 2012; Suchman et al., 2017). However, it is not clear, whether maternal prenatal PRF and AAI-RF are similarly associated with mother-infant interaction and substance use as intervention outcomes. This study examined, first, the interrelations and differences between mothers' prenatal AAI-RF and pre- and post-natal PRF, in a sample of treatment-enrolled mothers with SUD. Second, we examined, whether AAI-RF and PRF have different effects on the mother-infant interaction quality during the intervention (at the child age of 4 months) and for the post-intervention change in mother-infant interaction quality from 4 to 12 months. Finally, we examined, whether AAI-RF and PRF predict maternal substance use during the child's first year.

### Attachment System and Mother's Attachment-Related RF

Attachment is an evolutionary-based motivational system aimed at seeking security and protection from close relationships under distress (Bowlby, 1969). In infancy, attachment is displayed in the infant's proximity-seeking behavior toward the attachment figure (usually the parents; Bowlby, 1969), varying in quality based on parental sensitivity toward infant cues (Ainsworth, 1978). Attachment is also accompanied by information-processing models (internal working models), which are generalized representations regarding the availability of others and the competence and worthiness of self that guide interpersonal perception and behavior (Bowlby, 1969, 1980; Ainsworth, 1990). Securely attached children and adults openly express their emotional needs and are capable of relying both on themselves and others as a source of regulation and comfort (Ainsworth, 1978; Main et al., 1985). In adults, attachment can be measured from the narrative features of an interview regarding their childhood experiences, the Adult Attachment Interview (AAI; Main et al., 2003). A mother's own childhood attachment experiences become especially activated during pregnancy when there is an intensive psychological process regarding the preparation for motherhood (Wilson et al., 2007). Research shows that maternal prenatal attachment security is highly predictive of her sensitivity to the future child, and eventually, to the child's attachment security, thus leading to the *intergenerational transmission of attachment* (Main et al., 1985; van IJzendoorn, 1995; Benoit et al., 1997).

Fonagy et al. (1991) developed a specific RF scale to assess mothers' adult attachment-focused RF from the AAIs (AAI-RF) based on how well they were able to reflect upon their childhood relationships with their own parents. They found in their pivotal work that mothers with secure attachment showed higher AAI-RF, and it was specifically their RF capacity that predicted the intergenerational transmission of attachment (Fonagy et al., 1991). RF has its roots in the mother's own early attachment relationships: Children who are sensitively responded to and receive contingent mirroring and validation of affects, learn to use RF as a means of affect regulation (Gergely and Watson, 1999; Bateman and Fonagy, 2019). The capacity to use RF is thus strongly tied to an adult's capacity to regulate both their own (Fonagy et al., 2002; Ensink et al., 2016; Kivity et al., 2021) and their children's emotions and behavior (Smaling et al., 2017; Moser et al., 2019; Schultheis et al., 2019; Borelli et al., 2021).

Overall, maternal AAI-RF seems to be a factor with broad significance over both her own and her child's wellbeing. Problems in AAI-RF have been of interest in psychotherapy research and have been linked with mental health problems, such as depression (Fischer-Kern et al., 2013), and personality disorders (Nazzaro et al., 2017). Interestingly, parental AAI-RF also seems to be highly relevant for child development, including offspring mentalization (Rosso et al., 2015) and psychopathology (Esbjørn et al., 2013). Regarding parenting, research on AAI-RF is more scarce, but prenatal AAI-RF has been linked with post-natal parental sensitivity and mind-mindedness (parental ability to use mental state language), as well as child's secure attachment (Arnott and Meins, 2007; Ensink et al., 2016). High maternal prenatal AAI-RF seems to especially protect mothers from acting out negative (intrusive, aggressive, and withdrawn) caregiving behaviors and their children from developing disorganized attachment (Ensink et al., 2016). However, in the parenting context, most research during the recent years has concerned another related concept, *parenting-focused RF (PRF)*, implying parental RF in the current, ongoing relationship with her child. Below, we discuss PRF along with its theoretical framework, the maternal caregiving system.

### Caregiving System and Mother's Parenting-Related RF

Solomon and George (1996) described caregiving as another behavioral-motivational evolutionary system that emerges during pregnancy, forming a new psychological structure organized around the protection of the child. Like the attachment system, it is affected by the early relationships with one's parents but is also highly malleable to experiences with the individual child, including child characteristics. Similar to attachment, the caregiving system involves parallel information-processing models, i.e., caregiving representations. These comprise representations of the child and the self as a parent to that particular child that are formed already during pregnancy (Stern, 1995; Slade et al., 2009). Similarly to maternal attachment representations, her secure or balanced caregiving representations are known to be related to the mother's own secure attachment history, infant secure attachment, and higher mother-infant interaction quality (Vreeswijk et al., 2012; Fonseca et al., 2018).

Along these lines, Slade (2005) developed the concept of PRF to describe RF directly in the context of caregiving. They proposed that PRF is more relevant for intergenerational transmission of attachment than RF based on adults' reflections on their childhood experiences (AAI-RF). PRF is a capacity to interpret both the child's behaviors and one's parenting behavior as a function of underlying mental states, such as emotions, thoughts, and intentions. Highly reflective parents also view the child as an active agent (Sharp and Fonagy, 2008), with a mind of his/her own. Good PRF capacity is a prerequisite for the parent to correctly perceive, respond to and regulate the child's affects and behaviors, that is, to be sensitive.

Slade et al. developed an interview measure specific to PRF, Parent Development Interview (PDI; Slade et al., 2004), as well as its prenatal version Pregnancy Interview (PI; Slade, 2007). Research shows that higher maternal PRF (as measured with PDI) is associated with both maternal and child attachment security, secure/balanced caregiving representations, and higher mother-infant interaction quality (Slade et al., 2005b; Rostad and Whitaker, 2016; Zeegers et al., 2017; Alvarez-Monjarás et al., 2019; Alismail et al., 2021), as well as more optimal child socioemotional development (Nijssens et al., 2020). Already prenatally, higher maternal RF is predictive of her ability to form a close, affectionate bond with the baby-in-womb (Rohder et al., 2020) which is crucial for post-natal parenting (Foley and Hughes, 2018).

However, as pointed out by Camoirano (2017) in his review article on mentalization and parenting, research has not yet differentiated the interconnections and different outcomes of maternal PRF and AAI-RF. Although RF is considered a core mental capacity with generalized effects across different relationships, it is also partly relationship-specific, meaning it takes more effort to transfer it over different relationships (Fonagy et al., 2002; Ensink et al., 2019). Some evidence is available that AAI-RF and PRF are interrelated, but may reflect partially distinct functions of maternal attachment and caregiving systems. Two studies have found them to overlap: Crumbley (2009) showed in his doctoral dissertation a high correlation (0.53) between maternal prenatal AAI-RF and her PDI-RF at the infant age of 10 months, and (Ensink et al., 2019) found a similarly high correlation (0.51) between a mother's prenatal AAI-RF and her PRF at the infant age of 6 months. Concerning outcome-specificity, the latter study further showed that maternal PDI-RF, but not AAI-RF, was associated with maternal sensitivity at 6 months and child's attachment security at 16 months. However, it was unclear whether this was due to the concurrent measure of PRF and interaction quality, as only AAI-RF was measured prenatally, or to fundamental differences between AAI-RF and PRF. To sum it up, no previous study has examined the predictive role of maternal prenatal AAI-RF and PRF on post-natal outcomes, or in intervention contexts. In this study, we are interested in whether prenatal AAI-RF and PRF differentially predict parenting intervention outcomes in mothers with SUD, including post-natal RF, mother-infant interaction quality, and maternal substance use. Understanding these predictive effects already prenatally, when the relationship with the baby is still unfolding is vital for assessment, planning, and tailoring perinatal parenting interventions. Prenatally starting interventions are especially important for high-risk groups, such as mothers with SUD.

### Transition to Parenthood in Mothers With SUD

Pregnancy is a period of both opportunity and vulnerability for mothers with SUD (Van Scoyoc et al., 2016; Flykt et al., 2021). Treatment motivation is often enhanced by a strong desire to protect the baby (Van Scoyoc et al., 2016). During pregnancy, a mother's mind is also especially “open” to reorganizing previous attachment representations and creating new representations of the child and the self as a mother (Stern, 1995; Slade et al., 2009). Prenatal interventions thus have a unique potential to simultaneously reduce fetal substance exposure and help the mother emotionally bond with her baby, which in itself promotes abstinence (Massey et al., 2015; Flykt et al., 2021). However, pregnancy is also a period of heightened emotional upheaval and ambivalence, especially for mothers with SUD who often struggle to reorganize negative or traumatic childhood and couple relational experiences as part of their maternal identity (Söderström, 2012; Punamäki et al., 2013; Silva et al., 2013; Isosävi et al., 2016).

During pregnancy, mothers with SUD also need to deal with strong feelings of guilt, shame, and worry about harming the fetus with substances (Söderström, 2012; Silva et al., 2013). As substances may have previously been the primary means for emotion regulation, becoming abstinent during pregnancy may leave them increasingly vulnerable to difficult emotions. Further, mothers with SUD tend to show decreased capacity for RF both pre- and post-partum (Suchman et al., 2010; Pajulo et al., 2012; Smaling et al., 2015; Håkansson et al., 2018a), often impaired by a history of severe trauma (Håkansson et al., 2018b; Suardi et al., 2020). Difficulties in making sense of the emotional turmoil evoked by pregnancy and addiction also make it hard to form a close affective relationship with the baby, already evident during pregnancy in lower prenatal attachment to the fetus (Alhusen, 2008). Targeting maternal RF in substance use interventions has become especially central, as low RF is considered an underlying mechanism for both addiction and parenting difficulties (Macfie et al., 2020; Milligan et al., 2021).

### RF and Perinatal Treatments Outcomes for Mothers With SUD

Integrative interventions that address both substance use and parenting are the most effective forms of treatment for mothers with SUD (Niccols et al., 2012; Espinet et al., 2016). There is evidence that mentalizing-based interventions, that is, parenting interventions targeting maternal RF are especially useful. Ample evidence exists for the effectiveness of one such intervention, Mothering from the Inside Out (MIO; Suchman et al., 2010, 2016, 2017; Lowell et al., 2021). MIO is brief, supportive parenting psychotherapy that emphasizes strong therapeutic alliance and allows the mother to discuss recent stressful experiences, reflecting upon both their own and the child's emotions. The intervention has been shown to enhance maternal RF, abstinence, mental health, caregiving representations, and mother-child interaction quality, with improvements retained at follow-ups and positive child outcomes even growing stronger over time.

Similarly, in Finland, Pajulo et al. (2006, 2012) found that maternal RF increased as a result of residential treatment focused on decreasing parental substance use, improving parenting, especially maternal RF, and helping with practical issues. Further research evidence is available on mother-infant psychodynamic group therapy, applied as part of more comprehensive outpatient treatment for mothers with SUD in Finland (Belt and Punamäki, 2007; Belt et al., 2012; Punamäki and Belt, 2013). The intervention comprises 20-24 weekly 3-hour group sessions with two therapists, and another 3–6 months of individual follow-up meetings. In addition, one of the therapists is available by phone between sessions. The emphasis is on a safe therapeutic environment where the mothers first receive soothing and care that helps them be in touch with their own needs and emotions. They are then supported in responding to their infant's needs and understanding how their behavior affects the infant. The aim is also to encourage enjoyment from the child, parenting, and peer relations. Their past difficult life histories and emotions are reflected in the context of the current parent-child relationship, thus providing new experiences of secure attachment from the group and the therapists.

An earlier study regarding this mother-infant psychodynamic group therapy (Belt et al., 2012) showed that the treatment completion rate was high (84%) and it was effective in decreasing hostility and intrusiveness in mother-infant interaction. However, it was no more effective in improving other aspects of mother-infant interaction (e.g., sensitivity) or maternal mood or in decreasing substance use than individually tailored psychosocial parenting support (PSS, which represented treatment as usual). To our knowledge, there are no other studies on psychodynamic parent-infant psychotherapies among mothers with SUD, but among other high risks groups, parent-infant dyadic psychotherapy (Fonagy et al., 2016; Mattheß et al., 2021; Salomonsson et al., 2021) has been effective in improving parenting, maternal mood symptoms, and child socioemotional development.

Although the change in maternal RF seems to be an underlying mechanism for changes in mother-infant interaction (Lowell et al., 2021; Milligan et al., 2021), the role of pre-intervention RF has shown different dynamics across studies. Many studies show that high pre-intervention RF is beneficial for parenting intervention outcomes among high-risk mothers. Pajulo et al. (2012) showed that in mothers undergoing an inpatient SUD intervention, higher prenatal PRF predicted higher post-natal PRF (*R* = 0.56), and both low pre- and post-natal PRF predicted child's foster care risk. Slade et al. (2020) similarly showed in their high-risk mothers undergoing the Minding the baby-intervention that maternal prenatal PRF predicted higher post-natal PRF. On the contrary, Stacks et al. (2019) found that only those with the lowest pre-intervention PRF ( ≤ 3) showed increased PRF after an infant mental health home visiting intervention. This is in line with other findings that the most vulnerable individuals, such as those with trauma, attachment insecurity, or high psychological distress, may benefit more from parenting interventions than less vulnerable parents (Robinson and Emde, 2004; Paris et al., 2015; Stacks et al., 2019).

Research conducted with AAI-RF has mostly concerned adult psychotherapy patients, but the results are similarly inconclusive. In one study, patients with higher pre-treatment AAI-RF showed lower depressive symptoms after psychotherapy (Ekeblad et al., 2015). However, in another, Gullestad et al. (2013) found that patients with personality disorders who showed low pre-treatment AAI-RF had greater improvement in terms of psychosocial functioning in an outpatient psychotherapy treatment group. Interestingly, those with higher RF seemed to benefit faster, i.e., improved in their psychosocial functioning earlier in the treatment. Nonetheless, mother-infant interaction quality as a treatment outcome has rarely been studied in terms of the pre-intervention RF. The only study we are aware of, Pajulo et al. (2012), showed no association between maternal pre- or post-natal PRF and mother-infant interaction quality. Further, no previous study has differentiated the effects of prenatal PRF and AAI-RF.

### Research Questions

This study aims to explore the similarities and differences between maternal adult-attachment-focused RF (AAI-RF) and current-parenting-focused RF (PRF) during pregnancy, in terms of their role in treatment outcomes among mothers with SUD.

More specifically, the research questions and their associated hypotheses were the following:

(1) How are maternal prenatal AAI-RF and PRF associated with each other, and with maternal post-natal PRF? We hypothesize that: (a) both are significantly associated with each other and with post-natal PRF, and (b) maternal post-natal PRF is higher than prenatal AAI-RF and PRF.
(2) Do maternal prenatal AAI-RF and PRF predict the post-natal mother-infant interaction quality at the child age of 4 months (during intervention)? We hypothesize that: (a) higher AAI-RF and higher PRF both similarly predict higher maternal sensitivity and structuring; (b) PRF is more predictive of child responsiveness and involvement than AAI-RF; and (c) AAI-RF is more predictive of maternal regulatory-related aspects of interaction, hostility, and intrusiveness.
(3) Do maternal prenatal AAI-RF and PRF predict change in mother-infant interaction quality from 4 to 12 months (post-intervention)? We hypothesize that: (a) higher AAI-RF and PRF both similarly predict greater change in maternal sensitivity and structuring; (b) PRF is more predictive of change in child responsiveness and involvement than AAI-RF; (c) AAI-RF is more predictive of change in maternal hostility and intrusiveness.
(4) Do maternal prenatal AAI-RF and PRF predict maternal substance relapses during the child's first year? We hypothesize that lower prenatal AAI-RF and PRF similarly predict a higher risk for substance relapses.

## Materials and Methods

### Participants and Procedure

#### Main Sample

**Figure 1:**
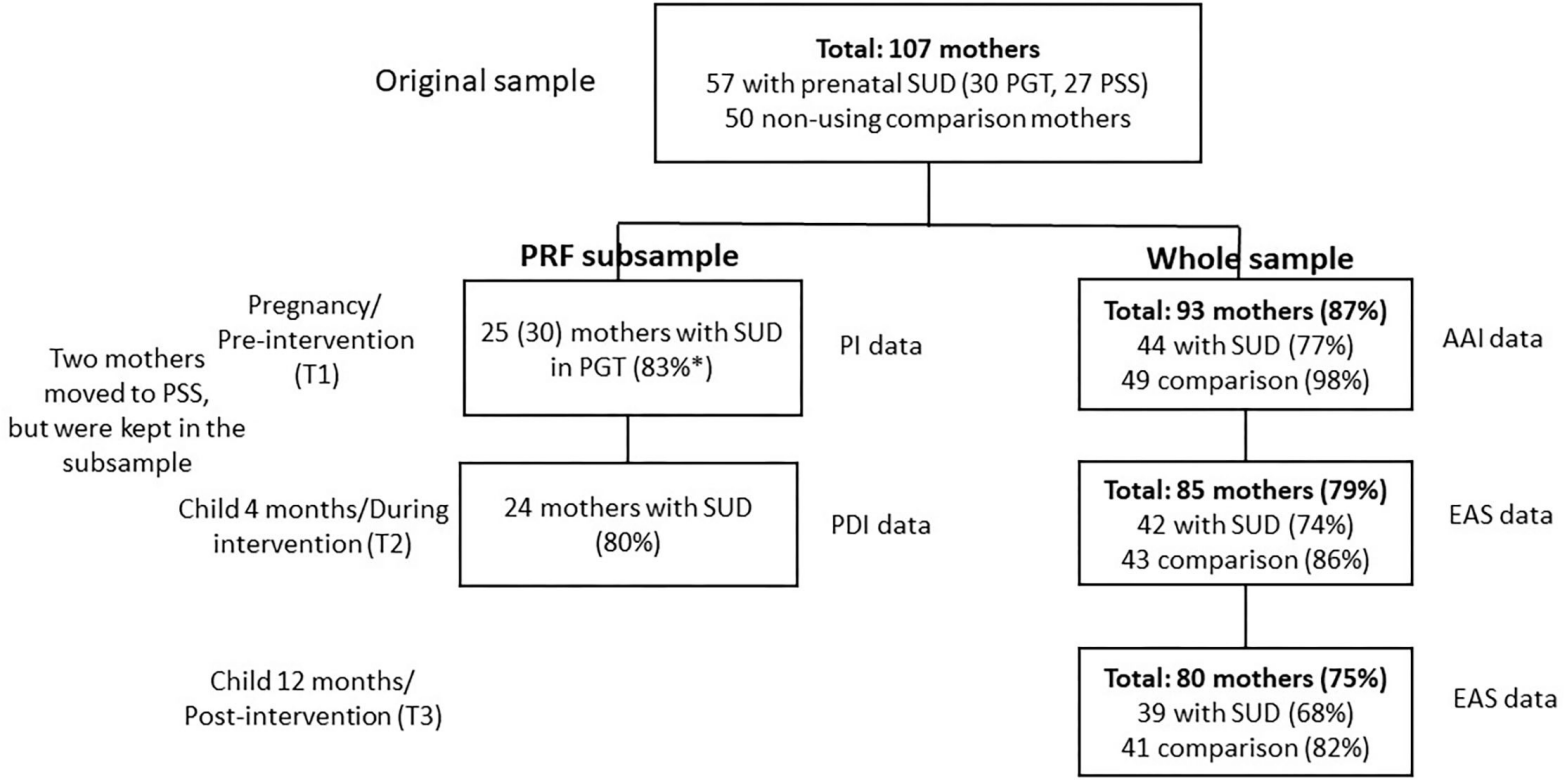
The participation flow chart of the study. SUD, substance use disorder; PGT, psychodynamic group therapy; PSS, Psychosocial support; PI, Pregnancy interview; PDI, Parent development interview; AAI, Adult Attachment Interview; EAS, Emotional Availability Scales. All mothers were included in the analyses based on intention to treat, and missing data was replaced with Multiple Imputation (MI). *30 mothers participated in PGT and had other data, but 5 had given birth at T1, so PI was not conducted for them and their data was imputed. Similarly, some mothers lacked prenatal AAI data, but were included in the study as they had other data. The rate of missingness was slightly higher for T2–T3 relapses than for T2 and T3 EA, 35 mothers with SUD (61%) reported whether they had had relapses.

Participants were 107 Finnish-speaking, Caucasian mothers and their infants (43.4% girls, 56.6% boys). Half of the mothers (*n* = 57) were recruited from two outpatient clinics in Southern Finland offering comprehensive, integrated substance use and parenting interventions (SUD group). The other half (*n* = 50) were low-risk comparison mothers recruited from hospital maternity clinics where they had contact due to pregnancy complications (e.g., gestational diabetes). The exclusion criteria for the comparison group were any lifetime illegal drug use or problematic alcohol use. Five comparison mothers had twins. Only data from one twin-child in each pair was used in the analyses (randomly selected). The participation flow chart of the study is presented in Figure 1.

All mothers in the SUD group had a diagnosis of drug-dependency and a history of more than 3 years of illicit drug use or polysubstance use. Most (80%) also self-reported using drugs intravenously, and more than half (58.8%) reported alcohol use above the clinical cut-off (≥3 for women) on the Alcohol Use Disorders Identification Test consumption scale (AUDIT-C).

In both clinics, the mothers received comprehensive, multi-professional support, including help from social services and addiction and mental health counseling. For parenting support, initially, 30 mothers were enrolled in psychodynamic mother-infant group psychotherapy (PGT), comprising 20–24 3-h weekly sessions, and a 3- to 6-month follow-up period of individual meetings. The rest (*n* = 27) were enrolled in intensive, individually tailored psychosocial parenting support (PSS), comprising meetings at the clinic or at home once or twice a week for 12 months. Two additional mothers initially from the PGT group changed into PSS, leading to *n* = 28 in the PGT and n = 29 in the PSS group (Interventions described in more detail in Belt and Punamäki, 2007; Belt et al., 2012; Punamäki and Belt, 2013). Most (87.2%) of the SUD group mothers reported stopping substance use during pregnancy and the rest reported diminishing the use. Almost a fourth (23.1%) had buprenorphine replacement therapy throughout pregnancy and four of their infants showed opiate withdrawal symptoms at birth.

#### PRF Subsample

Parenting-focused RF was measured only in the subsample of mothers in the PGT group, both pre-intervention and during the intervention (child age of 4 months). It was used as part of the therapeutic intervention, conducted by the therapists. Due to practical resources, these measures were not available for the PSS or comparison groups. We thus refer to the subsample or PRF subsample in this article, when we are describing the results regarding the PRF (Other measures were conducted for the whole sample). Participants of the subsample initially comprised 30 mothers and their infants (33.3% girls, 56.7% boys, information missing for 3 children). Most (*n* = 28) received psychodynamic group therapy. Two other mothers first started psychodynamic group therapy but changed to the psychosocial support intervention. They were, however, included in the subsample based on intention-to-treat and also because they had measures on all study variables (including post-natal PRF), and the two treatments were about similar in their effectiveness based on a previous study (Belt et al., 2012).

#### Procedure

The baseline measurement (T1), with questionnaires and interview measures, took place for most mothers during the third trimester of pregnancy, which was also pre-intervention for mothers in the two SUD groups. Two mothers in the PGT group and one in the PSS started the intervention already during the second trimester (gestational weeks 22–23). Five mothers in the PGT group and two in the PSS group started the intervention with a young baby, so prenatal RF measures were not conducted (they were replaced with data imputation, see Statistical analyses). The follow-ups were at the child age of 4 months (T2; for the SUD groups this was during the intervention) and 12 months (T3; for the SUD groups, this was post-intervention), including questionnaire and videotaped mother-infant interaction measures, and at T2, also with an interview measure for the PRF subsample of mothers.

### Measures

*Background variables* at T1 were reported with a questionnaire, including parity (primiparous vs. multiparous), mother's marital status (dichotomized into “married/cohabiting” vs. “single”), an education level (dichotomized into low, i.e., high school or lower vs. high, i.e., college or other education after high school) and economic difficulties (“Do you have difficulties paying bills” which was initially answered on a scale 1–5, 1 representing not at all and 5 representing extreme difficulties, and further dichotomized into Yes/No). At T2, the mothers further reported child sex and child health problems at birth (Yes/No).

*Maternal substance use* was measured at T1 by (a) alcohol use as measured with AUDIT consumption and dependence scales (range: 0–12; clinical cut-off ≥3 for consumption and ≥4 for dependence; Saunders et al., 1993); (b) a total number of drugs used [0–8, including cannabis, amphetamine, LSD, heroin, ecstasy, sniffing, medical misuse, or other drugs (which most often was an illegal use of buprenorphine)]; (c) whether they had intravenous use (Yes/no); (d) whether they received buprenorphine replacement therapy (Yes/no); (e) whether they experienced harm from their drug use (Yes/no); and (f) how psychologically and physically dependent they were of drugs (1–5, 1 representing not at all dependent and 5 completely dependent). At T1, they also reported changes in their drug use during pregnancy (all mothers reported either stopping or diminishing the use). Maternal post-natal use as indicated by self-reported drug relapses at T2 and T3 (combined and coded as No/Yes).

*Adult attachment-focused RF* was measured at T1 with Adult Attachment Interview (AAI; Main et al., 2003), and coded with the AAI-RF scale (Fonagy et al., 1998). AAI is a semi-structured interview that explores how individuals describe their childhood relationships to their primary caregivers, and how these experiences are considered to influence their developmental history and current personality. Audiotaped narratives were transcribed verbatim before coding. One trained, reliable coder coded all cases, and a second trained, reliable coder coded 20% of the sample. Inter-rater reliability (intra-class correlation) between the coders was 0.82. All differences were negotiated. The signs of mentalizing coded from the interviews can be divided into four categories: (a) the adult's awareness of the nature of different mental states; (b) the adult's clear and exact intention to understand the mental states underlying behavior; (c) the adult's ability to recognize a developmental aspect of mental states; and (d) the adult's ability to consider mental states in relation to the interviewer. The number of indications of true reflectiveness found in the transcribed narrative is the basis for assigning an overall score, ranging from −1 (negative RF) to 9 (exceptionally high RF).

*Parenting-focused RF* (PRF) was measured only in a subsample of mothers who started in psychodynamic group therapy, with two semi-structured interviews: at T1 with Pregnancy Interview (PI: Slade, 2007) and at T2 with Parent Development Interview (PDI: Slade, 2007), and coded with Addendums to Fonagy et al. (1998) RF scale (PDI Addendum: Slade et al., 2005a; PI Addendum: Slade et al., 2007). PI comprises 22 questions regarding mental states related to mothers' emotional experiences during pregnancy and their hopes, expectations, and fears about the future relationship with the child. PDI comprises 45 questions regarding the mental states related to maternal representations of their children, themselves as parents, and their relationships with the child. In evaluating both pre- and post-natal PRF, audiotaped narratives were transcribed verbatim before coding. The first author, who is a trained and reliable coder, coded all cases, and 17% were also coded by the fourth author, who is also a trained and reliable coder. The inter-rater reliability (intra-class correlation) for PI was 0.78 and for PDI, 1. All differences were negotiated. The signs of mentalizing are coded similarly to AAI-RF, but the coding manual specifically emphasizes how they are displayed regarding the child, parenthood, and parental relationship to the child. The number of indications of true reflectiveness found in the transcribed narrative is the basis for assigning an overall score, ranging from −1 (negative RF) to 9 (exceptionally high RF).

*Mother-infant interaction quality* was measured with Emotional Availability (EA) Scales (4th ed; Biringen, 2008). A 7–10 min free-play interaction was video-recorded at T2 and T3, either at home or at the clinic. The mothers were instructed to play as they usually would with their infants. The interaction was evaluated on four maternal scales: Sensitivity refers to the mother's adequate emotional and behavioral responses to the infant's cues. Structuring means the mother's ability to guide and scaffold the infant in developmentally appropriate ways. Non-intrusiveness indicates the maternal ability to be available without interfering with the infant's autonomy. Non-hostility is displayed in the maternal ability to refrain from harsh or impatient interactive behaviors. Further, two-child scales were used: Child responsiveness assesses the child's ability to respond to maternal emotional and behavioral interactive bids, and child involvement implies the extent to which the child invites the mother to interact with him/her. All tapes were coded by the first author, and 10% by the fourth author, who both are trained and reliable coders of EAS. Furthermore, 5% of additional tapes were jointly coded with the method developer. The inter-rater reliability ranged from 0.82–to 0.97. Differences were negotiated.

### Statistical Analyses

The associations between background and substance use variables with study variables were examined with chi-square tests and Student's *t*-tests, depending on whether the variables were continuous or categorical. The associations between background and substance use variables with maternal group status (for background variables: PGT, PSS, comparison; for substance use variables: PGT and PSS) were examined with chi-square tests and Student's *t*-tests (two-group comparisons) or Univariate ANOVAs (three-group comparisons). Analyses were conducted with SPSS version 28 for descriptive analyses and with Mplus version 8 for the main analyses.

For main analyses, missing data were replaced with Multiple Imputation (MI), using auxiliary variables, i.e., variables that are highly correlated with the imputed variables. This was done separately for the whole sample and the subsample of 30 mothers. We first examined the correlations between AAI-RF, PI-RF, and PDI-RF with Pearson's R, and the mean differences between the RF variables using Wald's tests. This was done only in the subsample, as PI-RF and PDI-RF were not measured for the whole sample. Second, we examined how AAI-RF and PI-RF predicted EA variables. The analyses were run separately for AAI-RF and PI-RF, as AAI-RF was examined in the whole sample (including both the SUD group and comparison group), and PI-RF only in the subsample of 30 mothers with SUD. Even though analyses between AAI-RF and EA were conducted with the whole sample, we further checked them in the subsample of 30 mothers. As there were few differences, we decided to use the whole sample when possible, and report only those results, when available. Analyses regarding AAI-RF (using the whole sample) were covaried with maternal substance use history (yes/no) and education level (low/high). Analyses concerning PI-RF were covaried with education level (group status was not covaried as there were no comparison mothers in this subsample). Finally, we examined whether AAI-RF and PI-RF predicted substance relapses during the child's first year, using linear regression. In the whole sample (regarding AAI-RF), analyses were performed only for substance-using mothers. Maternal education level was used as a covariate.

## Results

### Descriptive Statistics

Table 1 reports the study variable means and standard deviations for the two substance use groups (PGT and PSS) and the comparison group. The results showed that the two substance use groups differed from the comparison group in showing lower EA in all variables at T2, and in lower sensitivity, child responsiveness, and child involvement at T3. The PGT group also showed lower structuring than the comparison group at T3, whereas the PSS group did not differ from either group. There were no group differences in AAI-RF. Table 2 reports group differences in background variables, showing that mothers in both substance use groups had lower education levels and more financial difficulties than the comparison group, and were more often single, but did not differ from each other.

**Table 1:** Means, standard deviations, ranges, and group differences in study variables.

	**PGT**		**PSS**		**Comparison**			
	**M**	**SD**	**M**	**SD**	**M**	**SD**	**Range**	**F(df)**	***p***
PI-RF*	2.28	1.24					0–4 (−1–9)	
PDI-RF*	2.92	1.32					0–7 (−1–9)	
AAI-RF	3.92	0.35	3.61	0.36	3.57	0.24	PGT: 0–9; PSS: 1–7; comparison: 1–7 (−1–9)	0.35 (2,93)	0.71
T2 sensitivity	3.39^a^	0.25	3.13^a^	0.26	3.58^b^	0.18	2–7 (1–7)	13.70 (2,82)	**<0.001**
T2 structuring	3.82^a^	0.23	3.55^a^	0.24	4.64^b^	0.17	2–7 (1–7)	8.33 (2,82)	**<0.001**
T2 non-intrusiveness	3.43^a^	0.30	3.40^a^	0.31	4.67^b^	0.21	1–7 (1–7)	8.79 (2,82)	**<0.001**
T2 non-hostility	4.82^a^	0.27	5.00^a^	0.29	5.94^b^	0.20	2–7 (1–7)	7.05 (2,82)	**0.001**
T2 child responsiveness	3.34^a^	0.26	3.05^a^	0.28	4.47^b^	0.19	1–7 (1–7)	11.42 (2,82)	**<0.001**
T2 child involvement	3.34^a^	0.26	2.80^a^	0.27	3.98^b^	0.19	1–7 (1–7)	6.66 (2,82)	**0.002**
T3 sensitivity	4.05^a^	0.20	4.15^a^	0.23	5.04^b^	0.15	2–6.5 (1–7)	9.91 (2,77)	**<0.001**
T3 structuring	4.21^a^	0.20	4.32^ab^	0.22	4.89^b^	0.14	2.5–0.6.5 (1–7)	4.87 (2,77)	**0.01**
T3 non-intrusiveness	4.39	0.27	3.91	0.30	4.68	0.20	1.5–7 (1–7)	2.32 (2,77)	0.11
T3 non-hostility	5.25	0.24	5.03	0.27	5.56	0.18	2–7 (1–7)	1.50 (2,77)	0.23
T3 child responsiveness	3.96^a^	0.20	4.15^a^	0.23	4.99^b^	0.15	2.5–7 (1–7)	10.31 (2,77)	**<0.001**
T3 child involvement	3.86^a^	0.21	4.06^a^	0.24	4.78^b^	0.16	2–6.5 (1–7)	7.05 (2,77)	0.002
	**%**	***n***	**%**	***n***				**χ^2^** **(df)**	***p***
Substance relapses**								1.39 (1)	0.24
No	63.2	12	81.3	13					
Yes	38.6	7	18.7	3					

PGT, psychodynamic group therapy; PSS, psychosocial support. ^*^PI-RF and PDI-RF were measured only in the PGT group. ^**^Substance relapses were measured only in the PGT and PSS groups. Range refers to observed range (theoretical range in parentheses). F, Univariate Anova; df, degrees of freedom. ^a, b^Group means differ from each other significantly (p < 0.05) in Bonferroni post-hoc tests. Differences in n's are due to missing values.

*Bold values represent statistically significant group mean differences (p < 0.05)*.

**Table 2:** Group differences in background variables.

	**PGT**		**PSS**		**Comparison**			
	**%**	***n***	**%**	***n***	**%**	***n***	**χ^2^ (df)**	***p***
**Education level**							30.11 (2)	**<0.001**
Low	92%^a^	23	96%^a^	24	42.6%^b^	20		
High	8%^a^	2	4%^a^	1	57.4%^b^	27		
**Financial difficulties**							25.82 (2)	**<0.001**
Yes	63%^ab^	17	88%^a^	22	28%	14		
No	37%^ab^	10	12%^a^	3	72%	36		
**Marital status**							19.47 (2)	**<0.001**
Married or cohabiting	51.9%	14	62.5%	15	6%	3		
Single	48.1%	13	37.5%	9	94%	47		
**Parity**							0.71 (2)	0.70
Primiparous	38.5%	10	50%	12	46%	23		
Multiparous	61.5%	16	50%	12	54%	27		
**Child sex**							0.28 (2)	0.87
Girl	41.7%	10	42.9%	9	47.7%	21		
Boy	58.3%	14	57.1%	12	52.3%	23		
**Neonatal health problems**							2.10 (2)	0.35
Yes	25%	5	22.2%	4	11.6%	5		
No	75%	15	77.8%	14	88.4%	38		

*PGT, Psychodynamic group therapy; PSS, Psychosocial support; Low education, high school or less; High education, High school or more. ^a, b^Groups differ from each other based on standardized cell residuals. Differences in n's are due to missing values*.

*Bold values represent statistically significant group mean differences (p < 0.05)*.

Supplementary Table 1 shows the associations between background and study variables. Higher maternal education level was associated with higher AAI-RF, and higher T2 and T3 sensitivity, structuring, and child responsiveness, as well as T3 child involvement. More neonatal health problems were associated with the mother's substance relapses during the child's first year. There were no significant associations between marital status, economic problems, parity, or child sex and the study variables.

Regarding prenatal substance use variables, Table 3 shows that the two substance use groups differed in that PGT mothers reported higher alcohol consumption, whereas PSS mothers reported marginally more often (*p* = 0.055) receiving buprenorphine replacement therapy. Supplementary Table 2 further shows that those with higher reported prenatal alcohol consumption and alcohol dependence, a higher number of different drugs used and those reporting prenatal harm from drugs were more likely to show substance relapses during the child's first year. Those with intravenous use or reporting prenatal harm from drugs showed lower EA in all maternal and child dimensions. Instead, dyads with mothers undergoing buprenorphine replacement therapy showed higher T2 sensitivity, structuring, and child involvement. Those who stopped drug use during pregnancy had higher PI-RF and T2 sensitivity and child responsiveness than those who only diminished their use. Those reporting higher physical drug-dependence during pregnancy had less involving infants at T2, but curiously, showed less hostile interactive behaviors with their infant.

**Table 3:** Group differences in T1 substance use variables in mothers with SUD.

	**PGT**		**PSS**				
	**M**	**SD**	**M**	**SD**	**Range**	**t (df)**	***p***
AUDIT consumption	4.79	2.66	2.47	1.73	1–9 (0–12)	2.93 (32)	**0.006**
AUDIT dependence	1.38	1.31	1.14	1.11	0–4 (0–12)	0.64 (43)	0.53
Number of illegal drugs	5.07	2.46	4.54	2.32	0–8 (0–8)	0.81 (51)	0.42
Physical drug dependence	1.71	1.37	2.00	1.54	1–5 (1–5)	−0.68 (44)	0.50
Psychological drug dependence	2.67	1.49	2.23	1.51	1–5 (1–5)	0.99 (44)	0.33
	**%**	***n***	**%**	***n***		**χ^2^** **(df)**	***p***
**Harm from drugs**						1.37 (1)	0.24
Yes	17	73.9	12	57.1			
No	6	26.1	9	42.9			
**Intravenous use**						1.37 (1)	0.24
Yes	18	78.3	20	91.9			
No	5	21.7	2	9.1			
**Replacement therapy**						3.67 (1)	0.055
Yes	3	13	8	38.1			
No	20	87	13	61.9			
	**%**	***n***	**%**	***n***		**Fisher's exact test**
**Change during pregnancy**						0.11
Diminised use	16.7	4	0	0		
Stopped using	83.3	20	100	21		

*T1, Pregnancy (pre-intervention); PGT, Psychodynamic group therapy; PSS, psychosocial support. Range refers to the observed range of the variable in the sample. The theoretical range is reported in parentheses. df, degrees of freedom. Differences in n's are due to missing values*.

*Bold values represent statistically significant group mean differences (p < 0.05)*.

### The Associations and Differences Between Prenatal AAI-RF and PI-RF and Postnatal PDI-RF

Our first research question concerned the associations and mean level differences between maternal prenatal AAI-RF and PRF (PI-RF) and post-natal PRF (PDI-RF). The results (examined only in the subsample) indicated that all were highly inter-correlated, as hypothesized. AAI-PRF and PI-PRF were correlated at the level of *R* = 0.54, *p* = 0.004. PI-PRF and PDI-PRF were correlated at the level of *R* = 0.47, *p* = 0.005, whereas AAI-RF correlated with PDI-RF at the level of *R* = 0.56, *p* = 0.019. As hypothesized, maternal post-natal PDI-RF was higher than prenatal PI-RF, Wald (1) = 4.88, *p* = 0.027. Yet, contrary to hypotheses, post-natal PDI-RF level was lower than prenatal AAI-RF, Wald (1) = 4.68, *p 0*.03. PI-RF was also lower than AAI-RF, Wald (1) = 16.25, *p* < 0.001.

### The Associations Between Prenatal AAI-RF, PRF, and Mother-Child EA

Tables 4, 5 show the results for the second research question concerning the associations of prenatal AAI-RF and PI-RF with maternal and child T2 EA. The results partially supported our hypothesis that higher maternal prenatal PI-RF was associated with higher maternal structuring and child involvement. However, contrary to our hypothesis, there were no associations between prenatal AAI-RF and maternal EA. Substance use history was significant as a covariate, with mothers with SUD showing lower EA in all dimensions than comparison mothers.

**Table 4:** Associations between maternal prenatal AAI-RF and maternal and child EA at 4 months.

	**Maternal substance use (yes/no)**					**Maternal education level**					**Prenatal AAI-RF**					
	**B**	**β**	**S.E. (B)**	**95 CI% (B)**	***p***	**B**	**β**	**S.E. (B)**	**95 CI% (B)**	***p***	**B**	**β**	**S.E. (B)**	**95 CI% (B)**	***p***
Maternal sensitivity	−1.25	−0.47	0.32	[−1.87, −0.63]	**<0.001**	0.02	0.006	0.37	[−0.71, 0.74]	0.97	0.03	0.04	0.08	[−0.12, 0.18]	0.68
Maternal structuring	−0.89	−0.38	0.29	[−1.46, −0.31]	**0.003**	0.005	0.003	0.33	[−0.65, 66]	0.99	0.08	0.11	0.07	[−0.07, 0.22]	0.30
Maternal non-intrusiveness	−1.23	−0.41	0.35	[−1.92, 0.54]	**<0.001**	−0.01	−0.004	0.36	[−0.71, 0.68]	0.97	−0.08	−0.09	0.09	[−0.26, 0.11]	0.42
Maternal non-hostility	−1.16	−0.42	0.33	[−1.80, −0.53]	**<0.001**	−0.25	−0.08	0.37	[−0.97, 0.48]	0.51	0.04	0.05	0.07	[−0.10, 0.18]	0.59
Child responsiveness	−1.22	−0.44	0.35	[−1.90, −0.54]	**<0.001**	0.02	0.007	0.38	[−0.72, 0.76]	0.96	−0.001	−0.002	0.08	[−0.16, 0.15]	0.99
Child involvement	−0.91	−0.35	0.36	[−1.62, −0.20]	**0.012**	−0.11	−0.04	0.40	[−0.89, 0.67]	0.79	0.03	0.04	0.08	[−0.13, 0.19]	0.71

*AAI-RF, Mother's adult attachment-focused reflective functioning; EA, Emotional availability (in mother-infant interaction). As multiple imputation (MI) was used to replace missing data, we used the whole sample (n = 107) for the analyses*.

*Bold values represent statistically significant group mean differences (p < 0.05)*.

**Table 5:** Associations between maternal prenatal PRF and maternal and child EA at 4 months.

	**Maternal education level**					**Prenatal PRF**				
	**B**	**β**	**S.E. (B)**	**95 CI% (B)**	***p***	**B**	**β**	**S.E. (B)**	**95 CI% (B)**	***p***
Maternal sensitivity	−0.18	−0.04	0.57	[−1.29, 0.94]	0.76	0.23	0.28	0.18	[−0.12, 0.57]	0.20
Maternal structuring	−0.20	−0.05	0.33	[−0.84, 0.44]	0.53	0.32	0.46	0.13	[0.06, 0.59]	**0.016**
Maternal non-intrusiveness	−1.34	−0.18	0.84	[−2.97, 0.30]	0.11	−0.19	−0.14	0.31	[−0.80, 0.42]	0.54
Maternal non-hostility	−0.004	−0.002	1.66	[−3.25, 3.25]	1.00	0.04	0.03	0.24	[−0.45, 0.53]	0.87
Child responsiveness	−0.15	−0.03	0.63	[−1.38, 1.08]	0.81	0.25	0.27	0.22	[−0.18, 0.67]	0.25
Child involvement	−0.33	−0.08	0.59	[−1.48, 0.81]		0.33	0.42	0.15	[0.04, 0.63]	**0.028**

*PRF, Mother's current parenting-focused reflective functioning; EA, Emotional availability (in mother-infant interaction). As multiple imputations (MI) were used to replace missing data, we used the whole subsample for the analyses (n = 30)*.

*Bold values represent statistically significant group mean differences (p < 0.05)*.

Tables 6, 7 show the results for the third research question regarding the associations between prenatal AAI-RF and PI-RF and change in maternal and child EA from T2 to T3. The results were partially according to our hypotheses, showing that higher maternal AAI-RF predicted a decrease in maternal intrusiveness and hostility. Substance use history was significant as a covariate, with mothers with SUD showing more positive change in non-hostility than comparison mothers. Regarding prenatal PI-RF, contrary to our hypothesis, the results showed that lower PI-RF was associated with a more positive change in maternal structuring. Regarding covariates, higher maternal education level was associated with more positive change in maternal sensitivity, structuring, non-intrusiveness, child responsiveness, and child involvement.

**Table 6:** Associations between maternal prenatal AAI-RF and change in maternal and child EA from 4 to 12 months.

	**Maternal substance use (yes/no)**					**Maternal education level**					**Prenatal AAI-RF**				
	**B**	**β**	**S.E. (B)**	**95 CI%(B)**	***p***	**B**	**β**	**S.E. (B)**	**95 CI% (B)**	***p***	**B**	**β**	**S.E. (B)**	**95 CI% (B)**	***p***
Maternal sensitivity	0.42	0.16	0.38	[−0.33, 1.17]	0.27	0.19	0.07	0.44	[−0.67, 1.04]	0.67	0.07	0.10	0.10	[−0.12, 0.26]	0.46
Maternal structuring	0.32	0.13	0.35	[−0.35, 1.00]	0.35	0.06	0.02	0.41	[−0.73, 0.68]	0.88	−0.006	−0.008	0.09	[−0.19, 0.18]	0.95
Maternal non-intrusiveness	0.68	0.22	0.38	[−0.07, 1.43]	0.08	−0.07	−0.02	0.47	[−0.98, 0.85]	0.89	0.21	0.23	0.10	[0.02, 0.40]	**0.03**
Maternal non-hostility	0.86	0.30	0.32	[0.24, 1.49]	**0.007**	0.50	0.16	0.41	[−0.31, 1.32]	0.23	0.17	0.19	0.08	[0.004, 0.33]	**0.04**
Child responsiveness	0.38	0.14	0.42	[−0.43, 1.20]	0.36	0.12	0.04	0.47	[−0.79, 1.03]	0.80	0.02	0.02	0.11	[−0.21, 0.24]	0.90
Child involvement	0.16	0.06	0.44	[−0.69, 1.02]	0.71	0.22	0.07	0.50	[−0.75, 1.19]	0.65	0.01	0.02	0.11	[−0.21, 23]	0.91

*AAI-RF, Mother's adult attachment-focused reflective functioning; EA, Emotional availability (in mother-infant interaction). As multiple imputation (MI) was used to replace missing data, we used the whole sample (n = 107) for the analyses*.

*Bold values represent statistically significant group mean differences (p < 0.05)*.

**Table 7:** Associations between maternal prenatal PRF and change in maternal and child EA from 4 to 12 months.

	**Maternal education level**					**Prenatal PRF**				
	**B**	**β**	**S.E. (B)**	**95 CI% (B)**	***p***	**B**	**β**	**S.E. (B)**	**95 CI% (B)**	***p***
Maternal sensitivity	1.35	0.26	0.66	[0.05, 2.65]	**0.04**	−0.15	−0.15	0.20	[−0.54, 0.24]	0.45
Maternal structuring	1.69	0.33	0.81	[0.10, 3.28]	**0.04**	−0.39	−0.41	0.20	[−0.78, −0.006]	**0.046**
Maternal non-intrusiveness	3.07	0.42	0.94	[1.22, 4.91]	**0.001**	0.39	0.29	0.27	[−0.14, 0.91]	0.15
Maternal non-hostility	0.07	0.27	0.36	[−3.17, 3.76]	0.86	0.22	0.16	0.17	[−0.12, 0.51]	0.21
Child responsiveness	1.50	0.24	0.71	[0.12, 2.89]	**0.03**	−0.34	−0.29	0.26	[−0.86, 0.17]	0.19
Child involvement	1.84	0.29	0.76	[0.36, 3.32]	**0.02**	−0.38	−0.33	0.25	[−0.86, 0.10]	0.12

*PRF, Mother's current parenting-focused reflective functioning; EA, Emotional availability (in mother-infant interaction). As multiple imputations (MI) were used to replace missing data, we used the whole subsample for the analyses (n = 30)*.

*Bold values represent statistically significant group mean differences (p < 0.05)*.

### The Associations Between AAI-RF, PI-RF, and Maternal Substance Relapses

Our fourth research questions concerned the associations between maternal prenatal AAI-RF and PI-RF and maternal substance relapses during the child's first year. Our results indicated that lower PI-RF was associated with more substance relapses, B = −0.14, β = −0.38, S.E. (B) = 0.07, *p* = 0.044, 95% CI (B) = [−0.27, −0.003]. Maternal AAI-RF was not associated with substance relapses, B = 0.008, β = 0.008, S.E. (B) = 0.20, *p* = 0.97, 95% CI (B) = [−0.39, 0.40]. Maternal education level was not significant as a covariate (PRF model: B = 0.52, β = 0.27, S.E. (B) = 0.44, *p* = 0.23, 95% CI (B) = [−0.33, 1.38]; AAI-RF model: B = −0.15, β = −0.02, S.E.(B) = 1.35, *p* = 0.91, 95% CI(B) = [−2.79, 2.49].

## Discussion

In this study, we examined how maternal prenatal adult attachment-focused reflective functioning (AAI-RF) and current-parenting-focused reflective functioning (PRF) are associated with each other and post-natal PRF among treatment-enrolled mothers with SUD. We further examined how prenatal PRF and AAI-RF predict treatment outcomes, including post-natal mother-infant interaction and its changes during the intervention, and substance relapses during the child's first year. In accordance with our hypotheses, we found that prenatal AAI-RF and PRF were highly correlated with each other and with post-natal PRF. Importantly, these two types of RF, involving attachment and parenting, showed unique effects on early mother-infant interaction and interaction change in intervention, as well as on maternal abstinence. Only higher prenatal PRF was predictive of positive early maternal and child EA at 4 months (during the intervention), as well as maternal abstinence during the child's first year. Instead, both prenatal AAI-RF and PRF were important in predicting changes in interaction quality from 4 to 12 months. However, they had dimension-specific and opposite effects on maternal EA: Mothers with higher prenatal AAI-RF benefited from most of the interventions by showing a substantial decrease in negative interaction involving hostility and intrusiveness. Regarding PRF, it was mothers with lower prenatal PRF who showed increasing structuring in their interaction.

AAI-RF relates to maternal abilities in reflecting upon how they themselves were raised in their early relationships with parents, whereas prenatal PRF is a precursor of the actual parenting-related RF and is directed toward the baby-in-the-womb and one's developing mothering. PRF is considered parenting-specific and hence a more directly relevant form of RF for early parenting (Slade et al., 2005b), but few studies exist on prenatal PRF. AAI-RF is known to predict mental health intervention outcomes (Ekeblad et al., 2015), but has more rarely been studied in parenting contexts despite its high relevance for intergenerational transmission of attachment (Fonagy et al., 1991; Ensink et al., 2016).

Our first research question concerned the interrelations between maternal prenatal AAI-RF and pre- and post-natal PRF. In accordance with our hypothesis, all were highly inter-correlated. The level of associations was also comparable to earlier studies, with prenatal and post-natal PRF correlated at the level of *R* = 0.47 (*R* = 0.56 in Pajulo et al., 2012) and prenatal AAI-RF and post-natal PRF at the level of 0.56 (0.53 in Crumbley, 2009; and 0.51 in Ensink et al., 2019). Our findings broaden earlier studies in showing that similarly high levels of associations also exist for prenatal PRF and AAI-RF (*R* = 0.54). Hence, measuring different RF constructs during pregnancy seems to have theoretical and practical implications. Both give important information about future PRF with the child, yet probably through partly different dynamics. The AAI-RF develops in the context of the parent's own early family relationships that are specifically activated by pregnancy, whereas the prenatal PRF is based on representations of future parenting and caregiving of the child. Although both types of prenatal RF contain relatively little information about the actual relationship with the future child, they significantly correlated with the post-natal PRF, which is already highly impacted by the actual parenting experiences and child characteristics.

Similar to earlier findings (Suchman et al., 2010; Pajulo et al., 2012; Håkansson et al., 2018b) mothers with SUD generally showed very low levels of RF during pregnancy and post-partum in our sample. While the average level of RF in normative samples is 5, mothers with SUD in our sample showed an overall mean level below 4 for AAI-RF, and below 3 for pre- and post-natal PRF. Interestingly, prenatal AAI-RF levels were higher and showed more variability than prenatal PRF levels. AAI-RF ranged from what is considered to be very low/bizarre (0) to exceptionally high (9) for the substance-using women, whereas prenatal PRF ranged from very low/bizarre (0) to slightly below average (4). According to qualitative notes of the coders, mothers with SUD expressed few mental state reflections in their prenatal parenting interview. The identified reflections were present only among more highly reflective mothers, and tended to concern her substance use, its harmful effects on the child and mothering, and the potential difficulties in staying abstinent. Mothers with lower mentalization instead qualitatively showed denial and idealization when discussing their substance use. In all mothers, mental states reflections regarding the child in other contexts were lacking. It is possible that when still in the early phases of recovery from substances, even mothers with a generally good mentalizing capacity are not fully able to utilize their RF regarding the child. This was shown by the result that even mothers with high AAI-RF showed lower-than-average prenatal PRF. Although there was a significant positive change in the average PRF capacity from pregnancy to 4 months, the PRF capacity at 4 months was still lower than maternal prenatal AAI-RF capacity. Since we did not measure post-intervention PRF, it is unclear whether PRF would have risen to the same level as the AAI-RF after the intervention. However, our results suggest that in mothers with SUD, the general AAI-RF level is important to understand, as it may be a good indicator of her overall RF capacity. It is possible that a lowered AAI-RF capacity may indicate a more permanent impairment due to for example unresolved trauma. Prenatal PRF capacity may instead be temporarily lowered when simultaneously struggling with abstinence and becoming a mother.

Our second and third research questions regarded the effects of maternal prenatal AAI-RF and PRF on mother-infant interaction at 4 months and changes in the interaction from 4 to 12 months (post-intervention). It has been suggested that maternal AAI-RF would be an especially good indicator of maternal general-level regulatory abilities regarding their negative affect and behavior, as mothers with higher RF are able to mentally take a step back and recognize their emotions, before reacting in hostile or aggressive ways (Ensink et al., 2016, 2019). Along these lines, we hypothesized that high prenatal AAI-RF rather than PRF would especially predict maternal non-intrusiveness and non-hostility. This was partially substantiated, although not visible until post-intervention, when mothers with higher prenatal AAI-RF showed a more positive change from 4 to 12 months in maternal non-intrusiveness and non-hostility. This seemed to be specific for AAI-RF, but not PRF capacity and was evident both in substance-using and comparison mothers. Our study thus confirmed that mothers with higher AAI-RF especially benefitted from parenting interventions in terms of being more able to regulate their expression of negative emotions (hostility) and their controlling or intrusive interactive behaviors. Our results are thus in line with the notion that AAI-RF is a specific indicator of maternal regulatory capacity, perhaps as it reflects maternal unresolved trauma.

AAI-RF has overall been suggested to be a weaker indicator of parenting than PRF, as it takes more effort to transfer the ability across a different relationship (Fonagy et al., 2002; Ensink et al., 2019). This was also evident in our study in AAI-RF failing to show more comprehensive associations with maternal or child interaction involving also positive dimensions, such as maternal sensitivity or structuring or infant involvement. Although some previous studies (e.g., Ensink et al., 2016) have found AAI-RF to link with maternal sensitivity, similarly to our findings, Ensink et al. (2019) failed to find this association.

Regarding maternal PRF, the results partially supported our hypothesis that higher prenatal PRF is associated with early gains from parenting interventions in terms of higher structuring and child involvement evident already at the child age of 4 months (during the intervention). Maternal structuring and child EA have rarely been studied in dyads with maternal SUD or overall in combination with maternal RF, although it is plausible that interaction problems arise in these dimensions as well. Mothers with SUD often show problems in executive functioning (EF; Håkansson et al., 2018a) which may make it especially hard for them to guide and regulate child behavior in an organized way, i.e., to use structuring. EF problems are also highly linked with maternal RF problems (Håkansson et al., 2018a), and it is also possible that mothers with higher EF also have higher RF, helping them more quickly pick the skills to structure their infants. It is interesting that higher maternal prenatal PRF predicted higher child involvement, that is, how actively the child initiates and invites the mother to interact. Children with substance exposure may be more passive and withdrawing in interactions (Savonlahti et al., 2005), perhaps partially due to exposure and partially from lacking experiences of dyadic reciprocity due to maternal problems. Since PRF is associated with viewing the child as an active psychological agent with a mind of his/her own, it is interesting that PRF is especially associated with the child being more active with the mother. Interestingly, a recent study (Hakanen et al., 2019) similarly highlighted the role of another risk factor, maternal post-partum depression, on problems specifically in maternal structuring and infant involvement beyond other EA dimensions, suggesting that examining dimensions above and beyond sensitivity are important in high-risk studies.

Contrary to our hypothesis, there was no significant association between maternal prenatal PRF and maternal sensitivity. Even though the association between post-natal PRF and maternal sensitivity is well-validated (see for a meta-analysis, Zeegers et al., 2017), very little previous research exists on the role of prenatal PRF on maternal sensitivity overall, let alone on mothers with SUD. Interestingly, Smaling et al. (2016), the only study on *prenatal* PRF we are aware of, found a specific association between maternal prenatal PRF and sensitivity only in teaching situations, which may resemble more of our measure of structuring. One potential reason for this unexpected lack of findings was perhaps that the PGT intervention used in our study was not directly focused on increasing maternal sensitivity, lacking the most effective means, such as the use of video feedback (see van IJzendoorn et al., 2022). The current PGT interventions instead concentrated more around supporting maternal abilities to regulate themselves and their infants, which was also evident in more effectiveness for intrusiveness and hostility rather than sensitivity (Belt et al., 2012). It should, however, be noted that although the associations of prenatal PRF with maternal sensitivity and child responsiveness were not significant, they were in the same direction as with maternal structuring and child involvement. It is possible that with larger sample size, PRF could also significantly predict those dimensions.

Interestingly, and further highlighting the differences between maternal prenatal AAI-RF and PRF, while *higher* prenatal AAI-RF predicted more positive interaction change, it was *lower* prenatal PRF that predicted more positive interaction change, namely in maternal structuring. Earlier studies similarly show mixed results regarding whether less or more vulnerable individuals benefit more from parenting interventions. Some studies suggest, similarly to our PRF results, that more vulnerable individuals benefit more from parenting interventions, perhaps simply because they need them more (Robinson and Emde, 2004; Paris et al., 2015; Stacks et al., 2019). Actually, Stacks et al. (2019) even found that only mothers with low PRF (3 or below) received benefits from their early home-visiting intervention. In our sample, most mothers had prenatal PRF of three or below. Other studies have, however, found opposite effects: For example, Pajulo et al. (2012) showed that higher maternal prenatal PRF predicted lower risk for child foster care placements. Finally, some studies have even found similarly mixed effects, showing for example that both securely and insecurely attached parents benefit from parenting interventions, but in different ways (Cassidy et al., 2011; Berlin et al., 2018).

Our final research question concerned the predictive role of prenatal AAI-RF and PRF for maternal abstinence, indicated by the occurrence of substance relapses during the child's first year. In accordance with a previous study (Pajulo et al., 2012), lower maternal prenatal PRF was predictive of substance relapses during the child's first year, whereas AAI-RF was not. Addiction and parenting are considered neurobiologically intertwined: For instance, parental distress is known to increase substance relapses, and the substance-induced neurobiological alterations in maternal neural-hormonal processes (e.g., oxytocine and dopamine systems) also have harmful consequences on parenting (Rutherford and Mayes, 2019; Strathearn et al., 2019). This interconnection between addiction and parenting was visible even in our descriptive findings showing that besides RF, several of the mother's prenatal addiction characteristics predicted both substance relapses and mother-infant interaction.

### Limitations of the Study

The study was limited in its small sample size, especially in the subsample of mothers with whom maternal PRF was examined. Based on power analysis, we deem that the subsample study was able to identify medium-sized, but not small effects. The small sample size was due to maternal PRF measures being part of the PGT intervention and being conducted by the therapists, whereas practical resources hindered their use in other groups. The subsample was also limited in lacking any comparison group. It would have been ideal to conduct PI and PDI for the whole sample, as we did with other measures of the study. Now the results concerning only AAI-RF were conducted with a larger sample than the PRF results, although we did verify that they were similar also in the subsample. It would also have been interesting to look at maternal PRF post-treatment, to have more knowledge on whether prenatal RF predicted its change in treatment, and whether it eventually gained the same level as maternal prenatal AAI-RF. Further, maternal substance use was measured with self-report, whereas biological samples or registry information would have provided a more reliable report. Mothers did give urine samples during the treatment, but these were not accessible to researchers. However, it is convincing that most mothers were able to stop using substances at least at the end of pregnancy, as only a small amount of infants showed withdrawal symptoms at birth, and neonatal health was not different from the comparison group. However, no exact information on the amount and duration of exposure exists, which may limit the generalizability of the findings to more heavily using/heavily exposed mother-infant dyads. Finally, it would have been preferable to conduct the PI measure for all mothers during the third trimester of pregnancy, as this is an ideal time recommended by the measure developers (Slade et al., 2007). However, as the research was conducted as part of real-life interventions, mothers started the treatment at different time points, and conducted PI pre-treatment as part of the intervention.

### Implications for Research and Practice

Our study indicates that the two types of maternal prenatal RF, AAI-RF, and PRF, both have a vital but distinct role in mother-infant interaction and abstinence as intervention outcomes in substance-using mothers. The findings contribute to the importance of examining and treating maternal PRF already during pregnancy among high-risk substance-using mothers, as maternal prenatal PRF was predictive of their post-natal RF, mother-infant interaction, and abstinence. Our findings indicate that in the emotional turmoil of prenatal substance use, most if not all mothers struggle with developing a PRF capacity needed in forming a close affective relationship with the baby and one's maternal identity. However, mothers in our study showed much more varying levels of prenatal AAI-RF than PRF, including several mothers who were able to mentalize well about their own childhood experiences, but not about their future parenting. Although future studies with larger samples are needed, it is possible that prenatal AAI-RF is a more reliable indicator of the basic RF capacity in mothers with SUD, while PRF capacity may be more generally disturbed by the early-stage substance recovery. Clinically, as higher prenatal AAI-RF is an indicator of understanding and resolving one's own earlier, adverse and traumatic experiences, it may also suggest a better capacity to work with one's parenting under therapeutic settings, especially regarding the future regulatory capacity with the infant. Hence, it is also plausible that SUD mothers with lower prenatal AAI-RF are more traumatized and need more trauma-specific intervention elements in their treatment, to help them in their regulation and interaction with children. Assessing maternal AAI-RF may be one tool for the identification of mothers with such needs already during pregnancy.

Our results on the inter-connections between PRF, addiction, and mother-infant interaction quality also indicate that supporting good pre- and post-natal mother-child bond, including maternal RF, also enhances abstinence and vice versa. Children are a strong motivating force for mothers with SUD. Integrated treatments addressing both addiction and parenting, and allowing the children to be present in treatment are known to be superior to treatments focused on addiction alone (Pajulo et al., 2006; Neger and Prinz, 2015). Protecting children from the harm of substance use and learning to feel rewarded by interaction with the child, instead of substances, are at the core of perinatal substance use treatments (Van Scoyoc et al., 2016; Flykt et al., 2021).

Regarding future research needs, an important issue also emerged regarding the qualitative perceptions received from both AAI-RF and PRF main coders (who both are also experienced, psychotherapists). They independently noted that the interviews did not always seem to capture all clinically important phenomena, such as dissociative, aggressive, helpless, or role-reversing features. It is good to recognize that the current RF coding systems were developed for lower-risk populations. Parental RF system has indeed received criticism for how well it works with certain at-risk populations (e.g., Fonagy et al., 2016; Slade et al., 2022). Recent studies have already started to address these issues. Sleed et al. (2021) developed a new coding system, Assessment of representational risk (ARR), which qualitatively assesses maternal representational risk features from PDI, including hostile, helpless, and narcissistic features. Terry et al. (2021) applied the Hostile-Helpless coding system (initially developed for coding of attachment in high-risk populations, Lyons-Ruthn et al., 1995–2005), in coding high-risk PI's. We suggest that future studies could perhaps provide more clinically rich information if coded with such instruments specifically tailored for high-risk parents.

To conclude, mothers with prenatal SUD need treatments that are multi-professional and comprehensive and should be started during pregnancy to prevent both fetal exposure and the development of dyadic interaction problems (Flykt et al., 2021). The parenting component of treatments should ideally include the following elements: First, direct support of sensitivity toward the infant (such as with the use of video feedback, van IJzendoorn et al., 2022) is vital. Second, the mothers need help in mentalizing the infant and their parenting. Finally, support of the maternal own regulatory abilities is vital, including help in making sense of her difficult life experiences in a trauma-informed, respectful, and safe environment already starting from pregnancy.

## Data Availability Statement

The datasets analyzed for this study are available from the authors upon request. Participant privacy and ethical permissions related to this data do not allow public sharing of the data.

## Ethics Statement

The studies involving human participants were reviewed and approved by Tampere University Hospital Ethical Committee. Written informed consent to participate in this study was provided by the participating mothers on the behalf of themselves and their infants.

## Author Contributions

MF: writing, planning, analyses, and funding. RB: writing, planning, and data collection. SS: writing and analyses. MP: writing and planning. R-LP: writing, planning, and funding. All authors contributed to the article and approved the submitted version.

## Funding

The study was supported by a grant from Finnish Alcohol Foundation (Alkoholitutkimussäätiö).

## Conflict of Interest

The authors declare that the research was conducted in the absence of any commercial or financial relationships that could be construed as a potential conflict of interest.

## Publisher's Note

All claims expressed in this article are solely those of the authors and do not necessarily represent those of their affiliated organizations, or those of the publisher, the editors and the reviewers. Any product that may be evaluated in this article, or claim that may be made by its manufacturer, is not guaranteed or endorsed by the publisher.

## References

[B1] Ainsworth M. D. S. (1978). Patterns of Attachment: A Psychological Study of the Strange Situation. New York, NY: Lawrence Erlbaum.

[B2] Ainsworth M. D. S. (1990). “Some considerations regarding theory and assessment relevant to attachments beyond infancy,” in Attachment in the Preschool Years: Theory, Research and Intervention, eds M. T. Greenberg, K. Cicchetti, and E. M. Cummings (Chicago: University of Chicago Press), 463–488.

[B3] Alhusen J. L. (2008). A literature update on maternal-fetal attachment. J. Obstet. Gynecol. Neonatal Nurs. 37, 315–328. 10.1111/j.1552-6909.2008.00241.x 18507602 PMC3027206

[B4] Alismail F. Stacks A. M. Wong K. Brown S. Beeghly M. Thomason M. (2021). Maternal caregiving representations of the infant in the first year of life: associations with prenatal and concurrent reflective functioning. Infant Ment. Health J. 43, 311–327. 10.1002/imhj.21951 34879170 PMC9435997

[B5] Alvarez-Monjarás M. McMahon T. J. Suchman N. E. (2019). Does maternal reflective functioning mediate associations between representations of caregiving with maternal sensitivity in a high-risk sample? Psychoanal. Psychol. 36, 82–92. 10.1037/pap0000166 30853749 PMC6404967

[B6] Arnott B. Meins E. (2007). Links among antenatal attachment representations, postnatal mind-mindedness, and infant attachment security: a preliminary study of mothers and fathers. Bull. Menninger Clin. 71, 132–149. 10.1521/bumc.2007.71.2.132 17666003

[B7] Bateman A. Fonagy P. (2019). Handbook of Mentalizing in Mental Health Practice. Richmond, VA: American Psychiatric Publishing, Inc.

[B8] Belt R. Punamäki R.-L. (2007). Mother - infant group psychotherapy as an intensive treatment in early interaction among mothers with substance abuse problems. J. Child Psychother. 33, 202–220. 10.1080/00754170701437096

[B9] Belt R. H. Flykt M. Punamäki R.-L. Pajulo M. Posa T. Tamminen T. (2012). Psychotherapy groups and individual support to enhance mental health and early dyadic interaction among drug-abusing mothers. Infant Ment. Health J. 33, 520–534. 10.1002/imhj.21348 28520270

[B10] Benoit D. Parker K. C. H. Zeanah C. H. (1997). Mothers' representations of their infants assessed prenatally: stability and association with infants' attachment classifications. J. Child Psychol. Psychiatry 38, 307–313. 10.1111/j.1469-7610.1997.tb01515.x 9232477

[B11] Berlin L. J. Martoccio T. L. Jones Harden B. (2018). Improving early head start's impacts on parenting through attachment-based intervention: a randomized controlled trial. Dev. Psychol. 54, 2316–2327. 10.1037/dev0000592 30335427

[B12] Biringen Z. (2008). The Emotional Availability (EA) Scales (4th Edition): Infancy/Early Childhood Version. Boulder, CO. Available online at: www.emotionalavailability.com

[B13] Borelli J. L. Ensink K. Gillespie M. L. Falasiri E. Bernazzani O. Fonagy P.. (2021). Mothers' self-focused reflective functioning interacts with childhood experiences of rejection to predict current romantic relationship quality and parenting behavior. Fam. Process 60, 920–934. 10.1111/famp.12603 33026653

[B14] Bowlby J. (1969). Attachment and Loss. Volume 1, Attachment. New York, NY: Basic Books.

[B15] Bowlby J. (1980). Attachment and Loss. 3, Loss: Sadness and Depression. London: Basic Books.

[B16] Camoirano A. (2017). Mentalizing makes parenting work: a review about parental reflective functioning and clinical interventions to improve it. Front. Psychol. 8, 14. 10.3389/fpsyg.2017.00014 28163690 PMC5247433

[B17] Cassidy J. Woodhouse S. S. Sherman L. J. Stupica B. Lejuez C. W. (2011). Enhancing infant attachment security: an examination of treatment efficacy and differential susceptibility. Dev. Psychopathol. 23, 131–148. 10.1017/S0954579410000696 21262044

[B18] Conners N. A. Bradley R. H. Whiteside Mansell L. Liu J. Y. Roberts T. J. Burgdorf K.. (2004). Children of mothers with serious substance abuse problems: an accumulation of risks. Am. J. Drug Alcohol Abuse 30, 85–100. 10.1081/ADA-120029867 15083555

[B19] Crumbley A. H. (2009). The relationship specificity of the reflective function: an empirical investigation (Unpublised doctoral dissertation). The City University of New York, New York, NY, United States.

[B20] Ekeblad A. Falkenström F. Holmqvist R. (2015). reflective functioning as predictor of working alliance and outcome in the treatment of depression. J. Consult. Clin. Psychol. 84, 67–78. 10.1037/ccp0000055 26594944

[B21] Ensink K. Borelli J. L. Roy J. Normandin L. Slade A. Fonagy P. (2019). Costs of not getting to know you: lower levels of parental reflective functioning confer risk for maternal insensitivity and insecure infant attachment. Infancy 24, 210–227. 10.1111/infa.12263 32677198

[B22] Ensink K. Normandin L. Plamondon A. Berthelot N. Fonagy P. (2016). Intergenerational pathways from reflective functioning to infant attachment through parenting. Can. J. Behav. Sci. 48, 9–18. 10.1037/cbs0000030

[B23] Esbjørn B. H. Pedersen S. H. Daniel S. I. F. Hald H. H. Holm J. M. Steele H. (2013). Anxiety levels in clinically referred children and their parents: examining the unique influence of self-reported attachment styles and interview-based reflective functioning in mothers and fathers. Br. J. Clin. Psychol. 52, 394–407. 10.1111/bjc.12024 24117912

[B24] Espinet S. D. Motz M. Jeong J. J. Jenkins J. M. Pepler D. (2016). “Breaking the cycle” of maternal substance use through relationships: a comparison of integrated approaches. Addict. Res. Theory 24, 375–388. 10.3109/16066359.2016.1140148

[B25] Fischer-Kern M. Fonagy P. Kapusta N. D. Luyten P. Boss S. Naderer A.. (2013). Mentalizing in female inpatients with major depressive disorder. J. Nerv. Ment. Dis. 201, 202–207. 10.1097/NMD.0b013e3182845c0a 23407204

[B26] Flykt M. S. Salo S. Pajulo M. (2021). “A window of opportunity”: parenting and addiction in the context of pregnancy. Curr. Addict. Rep. 8, 578–594. 10.1007/s40429-021-00394-4

[B27] Foley S. Hughes C. (2018). Great expectations? Do mothers' and fathers' prenatal thoughts and feelings about the infant predict parent-infant interaction quality? A meta-analytic review. Dev. Rev. 48, 40–54. 10.1016/j.dr.2018.03.007

[B28] Fonagy P. Gergely G. Jurist E. L. Target M. (2002). Affect Regulation, Mentalization, and the Development of the Self. London: Other Press.

[B29] Fonagy P. Sleed M. Baradon T. (2016). Randomized controlled trial of parent-infant psychotherapy for parents with mental health problems and young infants. Infant Ment. Health J. 37, 97–114. 10.1002/imhj.21553 26939716

[B30] Fonagy P. Steele M. Steele H. Moran G. S. Higgitt A. C. (1991). The capacity for understanding mental states: the reflective self in parent and child and its significance for security of attachment. Infant Ment. Health J. 12, 201–218. 10.1002/1097-0355(199123)12:3<201::AID-IMHJ2280120307>3.0.CO;2-7

[B31] Fonagy P. Target M. Steele H. Steele M. (1998). Reflective-Functioning Manual, Version 5.0, for Application to Adult Attachment Interviews. London: University College London. 10.1037/t03490-000

[B32] Fonseca A. Nazaré B. Canavarro M. C. (2018). Mothers' and fathers' attachment and caregiving representations during transition to parenthood: an actor-partner approach. J. Reprod. Infant Psychol. 36, 246–260. 10.1080/02646838.2018.1449194 29553291

[B33] Frigerio A. Porreca A. Simonelli A. Nazzari S. (2019). Emotional availability in samples of mothers at high risk for depression and with substance use disorder. Front. Psychol. 10, 577. 10.3389/fpsyg.2019.00577 30936847 PMC6431618

[B34] Gergely G. Watson J. S. (1999). “Early socio–emotional development: contingency perception and the social-biofeedback model,” in Early Social Cognition: Understanding Others in the First Months of Life, ed P. Rochat (London: Lawrence Erlbaum Associates Publishers), 101–136.

[B35] Gullestad F. S. Johansen M. S. Høglend P. Karterud S. Wilberg T. (2013). Mentalization as a moderator of treatment effects: findings from a randomized clinical trial for personality disorders. Psychother. Res. 23, 674–689. 10.1080/10503307.2012.684103 22612470

[B36] Håkansson U. Söderström K. Watten R. Skårderud F. Øie M. G. (2018a). Parental reflective functioning and executive functioning in mothers with substance use disorder. Attach. Hum. Dev. 20, 181–207. 10.1080/14616734.2017.1398764 29105598

[B37] Håkansson U. Watten R. Söderström K. Skårderud F. Øie M. G. (2018b). Adverse and adaptive childhood experiences are associated with parental reflective functioning in mothers with substance use disorder. Child Abuse Negl. 81, 259–273. 10.1016/j.chiabu.2018.05.007 29775870

[B38] Hakanen H. Flykt M. Sinerv,ä E. Nolvi S. Kataja E.-L. Pelto J.. (2019). How maternal pre- and postnatal symptoms of depression and anxiety affect early mother-infant interaction? J. Affect. Disord. 257, 83–90. 10.1016/j.jad.2019.06.048 31299408

[B39] Isosävi S. Flykt M. Belt R. Posa T. Kuittinen S. Puura K.. (2016). Attachment representations among substance-abusing women in transition to motherhood: implications for prenatal emotions and mother-infant interaction. Attach. Hum. Dev. 18, 391–417. 10.1080/14616734.2016.1151904 26978721

[B40] Kivity Y. Levy K. N. Kelly K. M. Clarkin J. F. (2021). In-session reflective functioning in psychotherapies for borderline personality disorder: the emotion regulatory role of reflective functioning. J. Consult. Clin. Psychol. 89, 751–761. 10.1037/ccp0000674 34591548 PMC9634511

[B41] Lowell A. F. Peacock-Chambers E. Zayde A. DeCoste C. L. McMahon T. J. Suchman N. E. (2021). Mothering from the inside out: addressing the intersection of addiction, adversity, and attachment with evidence-based parenting intervention. Curr. Addict. Rep. 8, 605–615. 10.1007/s40429-021-00389-1 34306964 PMC8280593

[B42] Lyons-Ruth K. Melnick S. Yellin C. Atwood G. (1995–2005). Pervasively Unintegrated/Hostile-Helpless States of Mind on the Adult Attachment Interview (Unpublished Coding Manual). Cambridge, MA: Department of Psychiatry; Cambridge Hospital/Harvard Medical School.

[B43] Macfie J. Zvara B. J. Stuart G. L. Kurdziel-Adams G. Kors S. B. Fortner K. B.. (2020). Pregnant women's history of childhood maltreatment and current opioid use: the mediating role of reflective functioning. Addict. Behav. 102, 106134-. 10.1016/j.addbeh.2019.106134 31863966

[B44] Main M. Goldwyn R. Hesse E. (2003). Adult Attachment Scoring and Classification System (Unpublished Manuscript). University of California, Berkeley, CA, United States.

[B45] Main M. Kaplan N. Cassidy J. (1985). Security in infancy, childhood, and adulthood: a move to the level of representation. Monogr. Soc. Res. Child Dev. 50, 66–104. 10.2307/3333827

[B46] Massey S. H. Bublitz M. H. Magee S. R. Salisbury A. Niaura R. S. Wakschlag L. S.. (2015). Maternal–fetal attachment differentiates patterns of prenatal smoking and exposure. Addict. Behav. 45, 51–56. 10.1016/j.addbeh.2015.01.028 25644587 PMC4374036

[B47] Mattheß J. Eckert M. Becker O. Ludwig-Körner C. Kuchinke L. (2021). Potential efficacy of parent-infant psychotherapy with mothers and their infants from a high-risk population: a randomized controlled pilot trial. Pilot Feasibility Stud. 7, 210. 10.1186/s40814-021-00946-5 34819168 PMC8611874

[B48] Milligan K. Rodrigues E. R. Daari-Herman L. Urbanoski K. A. (2021). Parental reflective function in substance use disorder: individual differences and intervention potential. Curr. Addict. Rep. 9, 59–66. 10.1007/s40429-021-00391-7

[B49] Moser D. A. Suardi F. Rossignol A. S. Vital M. Manini A. Serpa S. R.. (2019). Parental reflective functioning correlates to brain activation in response to video-stimuli of mother–child dyads: links to maternal trauma history and PTSD. Psychiatry Res. Neuroimaging 293, 110985–110985. 10.1016/j.pscychresns.2019.09.005 31627112

[B50] Nazzaro M. P. Boldrini T. Tanzilli A. Muzi L. Giovanardi G. Lingiardi V. (2017). Does reflective functioning mediate the relationship between attachment and personality? Psychiatry Res. 256, 169–175. 10.1016/j.psychres.2017.06.045 28645076

[B51] Neger E. N. Prinz R. J. (2015). Interventions to address parenting and parental substance abuse: conceptual and methodological considerations. Clin. Psychol. Rev. 39, 71–82. 10.1016/j.cpr.2015.04.004 25939033 PMC4464898

[B52] Niccols A. Milligan K. Sword W. Thabane L. Henderson J. Smith A. (2012). Integrated programs for mothers with substance abuse issues: a systematic review of studies reporting on parenting outcomes. Harm. Reduct. J. 9, 14–14. 10.1186/1477-7517-9-14 22429792 PMC3325166

[B53] Nijssens L. Vliegen N. Luyten P. (2020). The mediating role of parental reflective functioning in child social–emotional development. J. Child Fam. Stud. 29, 2342–2354. 10.1007/s10826-020-01767-5

[B54] Pajulo M. Pyykkönen N. Kalland M. Sinkkonen J. Helenius H. Punamäki R.-L.. (2012). Substance-abusing mothers in residential treatment with their babies: importance of pre- and postnatal maternal reflective functioning. Infant Ment. Health J. 33, 70–81. 10.1002/imhj.20342 22899872 PMC3418818

[B55] Pajulo M. Suchman N. Kalland M. Mayes L. (2006). Enhancing the effectiveness of residential treatment for substance abusing pregnant and parenting women: focus on maternal reflective functioning and mother-child relationship. Infant Ment. Health J. 27, 448–465. 10.1002/imhj.20100 20119507 PMC2813060

[B56] Paris R. Herriott A. Holt M. Gould K. (2015). Differential responsiveness to a parenting intervention for mothers in substance abuse treatment. Child Abuse Negl. 50, 206–217. 10.1016/j.chiabu.2015.09.007 26455262

[B57] Punamäki R.-L. Belt R. (2013). “Psychoanalytic-attachment- oriented group intervention for substance-abusing mothers and their infants,” in Parenting and Substance Abuse, eds N. E. Suchman, M. Pajulo, and L. C. Mayes (Oxford: Oxford University Press). 10.1093/med:psych/9780199743100.003.0016

[B58] Punamäki R.-L. Belt R. Posa T. (2013). Emotions during the transition to parenthood among substance-abusing mothers: intensity, content and intervention effects. J. Reprod. Infant Psychol. 31, 222–244. 10.1080/02646838.2013.803046

[B59] Robinson J. L. Emde R. N. (2004). Mental health moderators of early head start on parenting and child development: maternal depression and relationship attitudes. Parent. Sci. Pract. 4, 73–97. 10.1207/s15327922par0401_4

[B60] Rohder K. Vaever M. S. Aarestrup A. K. Jacobsen R. K. Smith-Nielsen J. Schiotz M. L.. (2020). Maternal-fetal bonding among pregnant women at psychosocial risk: the roles of adult attachment style, prenatal parental reflective functioning, and depressive symptoms. PLoS ONE 15, e0239208. 10.1371/journal.pone.0239208 32941499 PMC7498041

[B61] Rosso A. M. Viterbori P. Scopesi A. M. (2015). Are maternal reflective functioning and attachment security associated with preadolescent mentalization? Front. Psychol. 6, 1134. 10.3389/fpsyg.2015.01134 26300824 PMC4523702

[B62] Rostad W. L. Whitaker D. J. (2016). The association between reflective functioning and parent–child relationship quality. J. Child Fam. Stud. 25, 2164–2177. 10.1007/s10826-016-0388-7

[B63] Rutherford H. J. V. Mayes L. C. (2019). Parenting stress: a novel mechanism of addiction vulnerability. Neurobiol. Stress 11, 100172. 10.1016/j.ynstr.2019.100172 31193862 PMC6543178

[B64] Salo S. Kivist,ö K. Korja R. Biringen Z. Tupola S. Kahila H.. (2009). Emotional availability, parental self-efficacy beliefs, and child development in caregiver-child relationships with buprenorphine-exposed 3-year-olds. Parent. Sci. Pract. 9, 244–259. 10.1080/15295190902844563

[B65] Salomonsson B. Kornaros K. Sandell R. Nissen E. Lilliengren P. (2021). Short-term psychodynamic infant–parent interventions at child health centers: outcomes on parental depression and infant social–emotional functioning. Infant Ment. Health J. 42, 109–123. 10.1002/imhj.21893 33155706

[B66] Saunders J. B. Aasland O. G. Babor T. F. de la Fuente J. R. Grant M. (1993). Development of the alcohol use disorders identification test (AUDIT): WHO collaborative project on early detection of persons with harmful alcohol consumption–II. Addiction. 88, 791–804. 10.1111/j.1360-0443.1993.tb02093 8329970

[B67] Savonlahti E. Pajulo M. Ahlqvist S. Helenius H. Korvenranta H. Tamminen T.. (2005). Interactive skills of infants with their high-risk mothers. Nord. J. Psychiatry 59, 139–147. 10.1080/08039480510022990 16195112

[B68] Schultheis A. M. Mayes L. C. Rutherford H. J. V. (2019). Associations between emotion regulation and parental reflective functioning. J. Child Fam. Stud. 28, 1094–1104. 10.1007/s10826-018-01326-z 31156323 PMC6538273

[B69] Sharp C. Fonagy P. (2008). The parent's capacity to treat the child as a psychological agent: constructs, measures and implications for developmental psychopathology. Soc. Dev. 17, 737–754. 10.1111/j.1467-9507.2007.00457.x

[B70] Silva S. A. Pires A. P. Guerreiro C. Cardoso A. (2013). Balancing motherhood and drug addiction: the transition to parenthood of addicted mothers. J. Health Psychol. 18, 359–367. 10.1177/1359105312443399 22544158

[B71] Slade A. (2005). Parental reflective functioning: an introduction. Attach. Hum. Dev. 7, 269–281. 10.1080/14616730500245906 16210239

[B72] Slade A. (2007). The Pregnancy Interview-Revised. New York: City College of New York.

[B73] Slade A. Aber J. L. Berger B. Bresgi I. Kaplan M. (2004). The Parent Development Interview—Revised (Unpublished manuscript). New York, NY: The City University of New York.

[B74] Slade A. Bernbach E. Grienenberger J. Levy D. Locker A. (2005a). Manual for Scoring Reflective Functioning on the Parent Development Interview. New York, NY: The City University of New York.

[B75] Slade A. Cohen L. J. Sadler L. S. Miller M. (2009). “The psychology and psychopathology of pregnancy: reorganization and transformation,” in Handbook of Infant Mental Health, ed C. H. Zeanah, Jr. (New York, NY: The Guilford Press), 22–39.

[B76] Slade A. Grienenberger J. Bernbach E. Levy D. Locker A. (2005b). Maternal reflective functioning, attachment, and the transmission gap: a preliminary study. Attach. Hum. Dev. 7, 283–298. 10.1080/14616730500245880 16210240

[B77] Slade A. Holland M. Ordway M. Carlson E. Jeon S. Close N. (2020) Minding the BabyⓇ: Enhancing parentalreflective functioning infant attachment in an attachment-based, interdisciplinary home visiting program. Develop. Psychopathol. 32, 123–137. 10.1017/S0954579418001463. 30636649

[B78] Slade A. Patterson M. Miller M. (2007). Addendum to Reflective Functioning Scoring Manual for Use With the Pregnancy Interview. New York: City College of New York.

[B79] Slade A. Sadler L.S. Eaves T. Webb D.L. (2022). Clinical Applications of the Pregnancy and Parent Development Interviews.

[B80] Sleed M. Isosävi S. Fonagy P. (2021). The assessment of representational risk (ARR): development and psychometric properties of a new coding system for assessing risk in the parent-infant relationship. Infant Ment. Health J. 42, 529–545. 10.1002/imhj.21932 34105777

[B81] Smaling H. J. Huijbregts S. C. Suurland J. Heijden K. B. Goozen S. H. Swaab H. (2015). Prenatal reflective functioning in primiparous women with a high-risk profile. Infant Ment. Health J. 36, 251–261. 10.1002/imhj.21506 25870154

[B82] Smaling H. J. A. Huijbregts S. C. J. Suurland J. van der Heijden K. B. Mesman J. van Goozen S. H. M.. (2016). Prenatal reflective functioning and accumulated risk as predictors of maternal interactive behavior during free play, the still-face paradigm, and two teaching tasks. Infancy 21, 766–784. 10.1111/infa.12137

[B83] Smaling H. J. A. Huijbregts S. C. J. van der Heijden K. B. Hay D. F. van Goozen S. H. M. Swaab H. (2017). Prenatal reflective functioning and development of aggression in infancy: the roles of maternal intrusiveness and sensitivity. J. Abnorm. Child Psychol. 45, 237–248. 10.1007/s10802-016-0177-1 27344154 PMC5241342

[B84] Söderström K. (2012). Mental preparation during pregnancy in women with substance addiction: a qualitative interview-study: mental preparation during pregnancy. Child Fam. Soc. Work 17, 458–467. 10.1111/j.1365-2206.2011.00803.x

[B85] Solomon J. George C. (1996). Defining the caregiving system: toward a theory of caregiving. Infant Ment. Health J. 17, 183–197. 10.1002/(SICI)1097-0355(199623)17:3<183::AID-IMHJ1>3.0.CO;2-Q

[B86] Stacks A. M. Barron C. C. Wong K. (2019). Infant mental health home visiting in the context of an infant—toddler court team: changes in parental responsiveness and reflective functioning. Infant Ment. Health J. 40, 523–540. 10.1002/imhj.21785 31095763

[B87] Stern D. N. (1995). The Motherhood Constellation: A Unified View of Parent-Infant Psychotherapy. New York, NY: Basic Books.

[B88] Strathearn L. Mertens C. E. Mayes L. Rutherford H. Rajhans P. Xu G.. (2019). Pathways relating the neurobiology of attachment to drug addiction. Front. Psychiatry 10, 737. 10.3389/fpsyt.2019.00737 31780957 PMC6857543

[B89] Suardi F. Moser D. A. Sancho Rossignol A. Manini A. Vital M. Merminod G.. (2020). Maternal reflective functioning, interpersonal violence-related posttraumatic stress disorder, and risk for psychopathology in early childhood. Attach. Hum. Dev. 22, 225–245. 10.1080/14616734.2018.1555602 30560713

[B90] Suchman N. E. DeCoste C. Leigh D. Borelli J. (2010). Reflective functioning in mothers with drug use disorders: implications for dyadic interactions with infants and toddlers. Attach. Hum. Dev. 12, 567–585. 10.1080/14616734.2010.501988 20931415 PMC2953729

[B91] Suchman N. E. DeCoste C. L. McMahon T. J. Dalton R. Mayes L. C. Borelli J. (2017). Mothering from the inside out: results of a second randomized clinical trial testing a mentalization-based intervention for mothers in addiction treatment. Dev. Psychopathol. 29, 617–636. 10.1017/S0954579417000220 28401850 PMC5407293

[B92] Suchman N. E. Ordway M. R. de las Heras L. McMahon T. J. (2016). Mothering from the inside out: results of a pilot study testing a mentalization-based therapy for mothers enrolled in mental health services. Attach. Hum. Dev. 18, 596–617. 10.1080/14616734.2016.1226371 27575343 PMC5102065

[B93] Terry M. Finger B. Lyons-Ruth K. Sadler L. S. Slade A. (2021). Hostile/Helpless maternal representations in pregnancy and later child removal: a pilot study. Infant Ment. Health J. 42, 60–73. 10.1002/imhj.21887 32816335

[B94] van IJzendoorn M. H. (1995). Adult attachment representations, parental responsiveness, and infant attachment: A meta-analysis on the predictive validity of the adult attachment interview. Psychol. Bull. 117, 387–403. 10.1037/0033-2909.117.3.387 7777645

[B95] van IJzendoorn M. H. Schuengel C. Wang Q. Bakermans-Kranenburg M. J. (2022). Improving parenting, child attachment, and externalizing behaviors: meta-analysis of the first 25 randomized controlled trials on the effects of video-feedback intervention to promote positive parenting and sensitive discipline. Dev. Psychopathol. 10.1017/S0954579421001462. [Epub ahead of print]. 35034668

[B96] Van Scoyoc A. Harrison J. A. Fisher P. A. (2016). Beliefs and behaviors of pregnant women with addictions awaiting treatment initiation. Child Adolesc. Soc. Work J. 34, 65–79. 10.1007/s10560-016-0474-0 31588163 PMC6777720

[B97] Vreeswijk C. M. Maas A. J. B. van Bakel H. J. (2012). Parental representations: a systematic review of the working model of the child interview. Infant Ment. Health J. 33, 314–328. 10.1002/imhj.20337 28520281

[B98] Wilson C. L. Rholes W. S. Simpson J. A. Tran S. (2007). Labor, delivery, and early parenthood: an attachment theory perspective. Pers. Soc. Psychol. Bull. 33, 505–518. 10.1177/0146167206296952 17400834

[B99] Zeegers M. A. J. Colonnesi C. Stams G.-J. J. M. Meins E. (2017). Mind matters: a meta-analysis on parental mentalization and sensitivity as predictors of infant-parent attachment. Psychol. Bull. 143, 1245–1272. 10.1037/bul0000114 28805399

