# Supplementary material for: Prenatal Reflective Functioning as a Predictor of Substance-Using Mothers' Treatment Outcome: Comparing Results From Two Different RF Measures
Source: Front Psychol. 2022 Jul 25;13:909414. doi: 10.3389/fpsyg.2022.909414 (PMC9359121; doi:10.3389/fpsyg.2022.909414)
Supplement: Supplementary file 1 [file Data_Sheet_1.docx]

Supplementary table 1: Associations between background and study variables

|  | Education level | | | | | | Economic problems | | | | | | Relationship status | | | |  | |
| --- | --- | --- | --- | --- | --- | --- | --- | --- | --- | --- | --- | --- | --- | --- | --- | --- | --- | --- |
|  | Low | | High | |  | | Yes | | No | |  | | Couple | | Single | |  | |
|  | M | SD | M | SD | t(df) | *p* | M | SD | M | SD | t(df) | *p* | M | SD | M | SD | t(df) | *p* |
| AAI-RF | 3.33 | 1.73 | 4.38 | 1.57 | -2.78(88) | **.007** | 3.76 | 1.85 | 3.59 | 1.54 | 0.48(93) | .63 | 3.72 | 1.61 | 3.50 | 2.04 | -0.51(93) | .61 |
| PI-RF^*^ | 2.25 | 1.11 | 3.50 | .71 | -1.53(20) | .14 | 2.13 | 1.25 | 2.67 | 1.22 | -1.02(22) | .32 | 2.57 | 1.34 | 2.00 | 1.05 | -1.12(22) | .28 |
| PDI-RF^*^ | 2.90 | 1.41 | 3.50 | .71 | -0.59(20) | .57 | 2.73 | 1.03 | 3.22 | 1.71 | -.088(22) | .39 | 3.26 | 1.36 | 2.58 | 1.24 | -1.26(22) | .22 |
| T2 sensitivity | 3.60 | 1.29 | 4.43 | 1.22 | -2.79(77) | **.007** | 3.65 | 1.32 | 4.20 | 1.31 | -1.90(83) | .06 | 4.02 | 1.34 | 3.61 | 1.36 | -1.19(82) | .24 |
| T2 structuring | 3.92 | 1.15 | 4.55 | 1.11 | -2.36(77) | **.021** | 4.04 | 1.15 | 4.30 | 1.21 | -1.04(83) | .30 | 4.20 | 1.18 | 4.03 | 1.24 | -0.56(82) | .58 |
| T2 non-intrusiveness | 3.83 | 1.54 | 4.43 | 1.35 | -1.72(77) | .09 | 3.88 | 1.63 | 4.22 | 1.38 | -1.04(83) | .30 | 4.09 | 1.44 | 3.92 | 1.80 | -0.43(82) | .67 |
| T2 non-hostility | 5.26 | 1.34 | 5.70 | 1.26 | -1.40(77) | .17 | 5.29 | 1.37 | 5.57 | 1.37 | -0.95(83) | .34 | 5.54 | 1.25 | 5.05 | 1.74 | -1.38(82) | .17 |
| T2 child responsiveness | 3.54 | 1.40 | 4.32 | 1.20 | -2.50(77) | **.015** | 3.55 | 1.49 | 4.13 | 1.21 | -1.98(83) | .052 | 3.92 | 1.40 | 3.58 | 1.35 | -0.93(82) | .36 |
| T2 child involvement | 3.33 | 1.34 | 3.82 | 1.20 | -1.60(77) | .11 | 3.33 | 1.33 | 3.73 | 1.26 | -1.42(83) | .16 | 3.54 | 1.33 | 3.53 | 1.31 | -0.06(82) | .95 |
| T3 sensitivity | 4.38 | 1.02 | 5.12 | .85 | -3.12(73) | **.003** | 4.46 | 1.10 | 4.68 | 1.02 | -0.92(78) | .36 | 4.66 | 1.05 | 3.32 | 1.04 | -1.17(77) | .25 |
| T3 structuring | 4.47 | .89 | 4.96 | .98 | -2.17(73) | **.033** | 4.55 | .93 | 4.61 | 1.00 | -0.25(78) | .80 | 4.62 | .95 | 4.53 | .96 | -0.35(77) | .73 |
| T3 non-intrusiveness | 4.40 | 1.27 | 4.70 | 1.18 | -0.98(73) | .33 | 4.39 | 1.43 | 4.48 | 1.12 | -0.29(78) | .78 | 4.46 | 1.27 | 4.44 | 1.29 | -0.05(77) | .96 |
| T3 non-hostility | 5.22 | 1.15 | 5.74 | .96 | -1.95(73) | .06 | 5.24 | 1.23 | 5.48 | 1.04 | -0.95(78) | .35 | 5.44 | 1.05 | 5.21 | 1.37 | -0.75(77) | .46 |
| T3 child responsiveness | 4.34 | 1.03 | 5.00 | .98 | -2.66(73) | **.01** | 4.45 | 1.06 | 4.60 | 1.05 | -0.63(78) | .53 | 4.63 | 1.06 | 4.21 | .95 | -1.48(77) | .14 |
| T3 child involvement | 4.21 | 1.07 | 4.82 | 1.02 | -2.36(73) | **.021** | 4.26 | 1.10 | 4.48 | 1.06 | -0.88(78) | .38 | 4.44 | 1.07 | 4.21 | 1.15 | -0.78(77) | .44 |
|  | % | n | % | n | χ^2^(df) | *p* | % | n | % | n | χ^2^(df) | *p* | % | n | % | n | χ^2^(df) | *p* |
| Substance relapses^**^ |  |  |  |  | 0.51(1) | .48 |  | |  | | 0.01(1) | .91 |  | |  | | 1.56(1) | .21 |
| Yes | 26.7 | 8 | 50 | 1 |  | | 28 | 7 | 30 | 3 |  | | 19 | 4 | 38.5 | 5 |  |  |
| No | 73.3 | 22 | 50 | 1 |  | | 72 | 18 | 70 | 7 |  | | 81 | 17 | 61.5 | 8 |  |  |
|  | Parity | | | |  | | Child sex | | | |  | | Neonatal health problems | | | |  | |
|  | Primiparous | | Multiparous | |  | | Girl | | Boy | |  | | Yes | | No | |  | |
|  | M | SD | M | SD | t(df) | *p* | M | SD | M | SD | t(df) | *p* | M | SD | M | SD | t(df) | *p* |
| AAI-RF | 3.39 | 1.32 | 3.88 | 1.96 | -1.39(91) | .17 | 3.79 | 1.79 | 3.89 | 1.57 | -0.27(81) | .79 | 3.58 | 1.98 | 3.83 | 1.54 | 0.48(74) | .63 |
| PI-RF^*^ | 2.00 | 1.26 | 2.58 | 1.24 | -1.12(21) | .28 | 2.50 | 1.20 | 2.23 | 1.30 | 0.47(19) | .64 | 1.80 | 2.05 | 2.54 | .88 | 1.10(16) | .29 |
| PDI-RF^**^ | 2.38 | 1.06 | 3.13 | 1.41 | -1.33(21) | .20 | 2.88 | .99 | 2.93 | 1.59 | -0.09(20) | .93 | 2.00 | 1.22 | 3.07 | 1.39 | 1.53(18) | .14 |
| T2 sensitivity | 3.82 | 1.30 | 4.06 | 1.40 | -0.77(80) | .45 | 4.01 | 1.28 | 3.86 | 1.39 | 0.52(83) | .61 | 3.58 | 1.46 | 4.06 | 1.32 | 1.18(78) | .24 |
| T2 structuring | 4.00 | 1.17 | 4.34 | 1.21 | -1.30(80) | .20 | 4.24 | 1.14 | 4.12 | 1.23 | 0.46(83) | .65 | 3.92 | 1.37 | 4.25 | 1.19 | 0.88(78) | .38 |
| T2 non-intrusiveness | 3.97 | 1.45 | 4.14 | 1.59 | -0.51(80) | .61 | 4.12 | 1.32 | 4.00 | 1.66 | 0.36(83) | .72 | 3.73 | 1.51 | 4.19 | 1.52 | 1.01(78) | .32 |
| T2 non-hostility | 5.39 | 1.45 | 5.43 | 1.34 | -0.13(80) | .89 | 5.49 | 1.36 | 5.38 | 1.39 | 0.35(83) | .73 | 5.15 | 1.68 | 5.50 | 1.30 | 0.84(78) | .41 |
| T2 child responsiveness | 3.73 | 1.39 | 3.96 | 1.41 | -0.73(80) | .47 | 3.91 | 1.30 | 3.79 | 1.45 | 0.40(83) | .69 | 3.65 | 1.63 | 3.95 | 1.33 | 0.70(78) | .49 |
| T2 child involvement | 3.43 | 1.45 | 3.64 | 1.22 | -0.72(80) | .47 | 3.50 | 1.23 | 3.56 | 1.38 | -0.22(83) | .82 | 3.19 | 1.64 | 3.66 | 1.24 | 1.19(78) | .24 |
| T3 sensitivity | 4.41 | 1.14 | 4.68 | 1.00 | -1.14(77) | .26 | 4.64 | .98 | 4.53 | 1.17 | 0.46(74) | .65 | 4.29 | 1.04 | 4.59 | 1.27 | 0.89(69) | .38 |
| T3 structuring | 4.45 | .98 | 4.65 | .95 | -0.90(77) | .37 | 4.71 | .93 | 4.48 | 1.02 | 1.04(74) | .30 | 4.25 | 1.16 | 4.61 | .95 | 1.15(69) | .25 |
| T3 non-intrusiveness | 4.14 | 1.22 | 4.63 | 1.28 | -1.73(77) | .09 | 4.65 | 1.12 | 4.29 | 1.40 | 1.25(74) | .22 | 4.13 | 1.46 | 4.45 | 1.23 | 0.80 (69) | .42 |
| T3 non-hostility | 5.11 | 1.10 | 5.53 | 1.14 | -1.67(77) | .10 | 5.38 | 1.11 | 5.39 | 1.20 | -0.05(74) | .96 | 5.04 | 1.16 | 5.40 | 1.17 | 0.96(69) | .34 |
| T3 child responsiveness | 4.39 | 1.10 | 4.61 | 1.02 | -0.89(77) | .38 | 4.72 | .93 | 4.40 | 1.13 | 1.35(74) | .18 | 4.46 | 1.16 | 4.54 | 1.04 | 0.25(69) | .80 |
| T3 child involvement | 4.23 | 1.10 | 4.47 | 1.07 | -0.97(77) | .33 | 4.61 | .95 | 4.20 | 1.16 | 1.68(74) | .10 | 4.21 | 1.14 | 4.38 | 1.06 | 0.51(69) | .61 |
|  | % | n | % | n | χ^2^(df) | *p* | % | n | % | n | χ^2^(df) | *p* | % | n | % | n | χ^2^(df) | *p* |
| Substance relapses^**^ |  | |  | | 0.58(1) | .45 |  | |  | | 1.12(1) | .29 |  | |  | | 6.35(1) | **.012** |
| Yes | 21.4 | 3 | 33.3 | 7 |  | | 21.4 | 3 | 38.9 | 7 |  | | 71.4 | 5 | 20.8 | 5 |  | |
| No | 78.6 | 11 | 66.7 | 14 |  | | 78.6 | 11 | 61.1 | 11 |  | | 28.6 | 2 | 79.2 | 19 |  | |

Note. ^*^Measured only in the PGT group. ^**^Measured only in the substance use groups. Low education=high school or less, High education=High school or more. df= degrees of freedom. p-values significant on the level of *p* <. 05 are bolded.

Supplementary table 2: Associations between T1 substance use variables and study variables.

|  | AUDIT consumption | | | | AUDIT dependence | | | | |  | Number of drugs | | | | Physical drug dependence | | | | Psychological drug dependence | | | |
| --- | --- | --- | --- | --- | --- | --- | --- | --- | --- | --- | --- | --- | --- | --- | --- | --- | --- | --- | --- | --- | --- | --- |
|  | R | | *p* | |  | R | | *p* | |  | R | | *p* | | R | | *p* | | R | | *p* | |
| AAI-RF | .11 | | .54 | |  | .20 | | .22 | |  | .14 | | .35 | | .16 | | .33 | | .02 | | .92 | |
| PI-RF^*^ | -.37 | | .14 | |  | -.10 | | .66 | |  | .15 | | .48 | | -.16 | | .50 | | -.21 | | .37 | |
| PDI-RF^*^ | .21 | | .41 | |  | -.06 | | .80 | |  | -.14 | | .52 | | -.06 | | .79 | | .25 | | .27 | |
| T2 sens. | -.08 | | .67 | |  | -.05 | | .78 | |  | -.006 | | .97 | | -.13 | | .43 | | -.18 | | .27 | |
| T2 struct. | .009 | | .96 | |  | -.04 | | .80 | |  | -.06 | | .72 | | -.23 | | .15 | | -.22 | | .18 | |
| T2 non-intr. | -.19 | | .33 | |  | -.07 | | .69 | |  | -.11 | | .50 | | .22 | | .19 | | .13 | | .42 | |
| T2 non-host. | -.002 | | .99 | |  | .05 | | .75 | |  | -.14 | | .37 | | .33 | | **.04** | | .19 | | .24 | |
| T2 child resp. | -.04 | | .85 | |  | -.12 | | .46 | |  | .002 | | .99 | | -.26 | | .11 | | -.20 | | .21 | |
| T2 child involvement | .02 | | .92 | |  | -.18 | | .28 | |  | -.009 | | .96 | | -.36 | | **.02** | | -.18 | | .27 | |
| T3 sens. | -.14 | | .50 | |  | .20 | | .24 | |  | -.24 | | .14 | | -.11 | | .54 | | .16 | | .36 | |
| T3 struct. | -.22 | | .27 | |  | .12 | | .50 | |  | -.02 | | .89 | | -.14 | | .42 | | .06 | | .74 | |
| T3 non-intr. | -.10 | | .61 | |  | .22 | | .20 | |  | -.05 | | .75 | | -.11 | | .52 | | .20 | | .25 | |
| T3 non-host. | -.28 | | .16 | |  | .04 | | .84 | |  | .11 | | .49 | | -.06 | | .73 | | .17 | | .33 | |
| T3 child resp. | -.28 | | .16 | |  | .11 | | .51 | |  | -.24 | | .15 | | -.09 | | .59 | | -.005 | | .98 | |
| T3 child involvement | -.19 | | .35 | |  | .19 | | .28 | |  | -.25 | | .12 | | -.13 | | .47 | | .01 | | .95 | |
|  | M | SD | t(df) | *p* | M | | SD | t(df) | *p* | M | | SD | t(df) | *p* | M | SD | t(df) | *p* | M | SD | t(df) | *p* |
| Substance relapses |  |  | -3.63(43) | **.001** |  | | | -2.77(64) | **.007** |  | | | 3.10(73) | **.003** |  |  | -1.83(62) | .07 |  | | -1.85(62) | .07 |
| Yes | 5.57 | 2.70 |  |  | 1.67 | | 1.32 |  |  | 5.00 | | 2.62 |  |  | 2.11 | 1.54 |  |  | 2.44 | 1.33 |  |  |
| No | 2.23 | 1.91 |  |  | 0.65 | | .97 |  |  | 2.06 | | 2.82 |  |  | 1.38 | 1.03 |  |  | 1.62 | 1.22 |  |  |

|  | Harm from drugs | | | | | | Intravenous use | | | | | | | Replacement therapy | | | | | | | | Change in substance use during pregnancy | | | | | | | | | | |
| --- | --- | --- | --- | --- | --- | --- | --- | --- | --- | --- | --- | --- | --- | --- | --- | --- | --- | --- | --- | --- | --- | --- | --- | --- | --- | --- | --- | --- | --- | --- | --- | --- |
|  | Yes | | No | |  | | Yes | | No | |  | | | Yes | | No | | | |  | | Diminished | | | | | Stopped | | | |  | |
|  | M | SD | M | SD | t(df) | *p* | M | SD | M | SD | t(df) | | *p* | M | SD | M | | SD | | t(df) | *p* | M | | | SD | | M | | | SD | t(df) | *p* |
| AAI-RF | 4.14 | 1.98 | 3.77 | 1.63 | -.87(70) | .39 | 3.65 | 1.84 | 4.10 | 1.68 | 1.10(72) | | .27 | 4.18 | 2.09 | 3.81 | | 1.70 | | -.65(71) | .52 | 4.00 | | | 3.74 | | 3.86 | | | 1.78 | .13(39) | .90 |
| PI-RF^*^ | 2.38 | 1.39 | 2.57 | 1.13 | .31(18) | .76 | 2.35 | 1.32 | 3.00 | 1.00 | 0.80(18) | | .43 | 3.00 | 1.41 | 2.39 | | 1.29 | | -.63(18) | .54 | 0.00 | | | 0.00 | | 2.60 | | | 1.14 | -2.22(19) | **.04** |
| PDI-RF^*^ | 3.13 | 1.36 | 2.00 | 1.22 | -1.65(19) | .12 | 2.65 | 1.06 | 3.75 | 2.36 | 1.47(19) | | .16 | 2.00 | .00 | 2.83 | | 1.50 | | -.19(19) | .85 | 3.00 | | | 1.15 | | 2.83 | | | 1.42 | .22(20) | .83 |
| T2 sens. | 3.19 | 1.02 | 4.44 | 1.31 | 4.24(69) | **<.001** | 3.17 | .92 | 4.48 | 1.38 | 4.61(71) | | **<.001** | 3.10 | .97 | 4.03 | | 1.37 | | 2.06(71) | **.04** | 2.00 | | | 0.00 | | 3.33 | | | .96 | -2.74(38) | **.009** |
| T2 struct. | 3.64 | .97 | 4.56 | 1.18 | 3.41(69) | **.001** | 3.55 | .93 | 4.63 | 1.17 | 4.30(71) | | **<.001** | 3.45 | .86 | 4.27 | | 1.21 | | 2.06(71) | **.04** | 3.25 | | | .87 | | 3.67 | | | .95 | -.84(38) | .41 |
| T2 non-intr. | 3.38 | 1.42 | 4.45 | 1.40 | 3.15(69) | **.002** | 3.33 | 1.41 | 4.50 | 1.38 | 3.57(71) | | **.001** | 3.60 | 1.60 | 4.05 | | 1.49 | | .88(71) | .38 | 2.38 | | | 1.70 | | 3.40 | | | 1.49 | -1.29(38) | .20 |
| T2 non-host. | 4.93 | 1.40 | 5.88 | 1.15 | 3.14(69) | **.002** | 4.84 | 1.48 | 5.91 | 1.07 | 3.58(71) | | **.001** | 5.05 | 1.42 | 5.51 | | 1.36 | | .98(71) | .33 | 4.25 | | | 1.44 | | 4.88 | | | 1.43 | -.83(38) | .41 |
| T2 child resp. | 3.23 | 1.17 | 4.26 | 1.43 | 3.16(69) | **.002** | 3.14 | 1.06 | 4.34 | 1.44 | 3.94(71) | | **<.001** | 3.05 | 1.12 | 3.94 | | 1.43 | | 1.87(71) | .07 | 1.63 | | | .48 | | 3.32 | | | 1.04 | -3.20(38) | **.003** |
| T2 child inv. | 3.16 | 1.11 | 3.83 | 1.40 | 2.12(69) | **.038** | 3.03 | 1.03 | 3.91 | 1.41 | 2.97(71) | | **.004** | 2.65 | 1.06 | 3.67 | | 1.32 | | 2.32(71) | **.02** | 2.50 | | | 1.08 | | 3.13 | | | 1.08 | -1.10(38) | .28 |
| T3 sens. | 3.94 | .98 | 4.94 | .92 | 4.18(64) | **<.001** | 4.00 | .86 | 5.00 | .99 | 4.37(65) | | **<.001** | 4.13 | 1.13 | 4.61 | | 1.04 | | 1.23(65) | .22 | 3.88 | | | 1.44 | | 4.03 | | | .83 | -.33(35) | .75 |
| T3 struct. | 4.10 | .85 | 4.94 | .89 | 3.81(64) | **<.001** | 4.27 | .86 | 4.86 | .96 | 2.65(65) | | **.01** | 4.63 | .83 | 4.59 | | .98 | | -.09(65) | .93 | 4.25 | | | .96 | | 4.21 | | | .82 | .09(35) | .93 |
| T3 non-intr. | 3.90 | 1.36 | 4.76 | 1.10 | 2.83(64) | **.006** | 4.12 | 1.31 | 4.70 | 1.18 | 1.92(65) | | .06 | 4.81 | 1.85 | 4.39 | | 1.18 | | -.89(65) | .38 | 3.38 | | | 1.49 | | 4.21 | | | 1.29 | -1.21(35) | .23 |
| T3 non-host. | 4.98 | 1.20 | 5.71 | .97 | 2.73(64) | **.008** | 5.17 | 1.16 | 5.62 | 1.04 | 1.69(65) | | .10 | 5.56 | 1.21 | 5.40 | | 1.11 | | -.39(65) | .70 | 5.00 | | | 1.41 | | 5.18 | | | 1.17 | -.29(35) | .78 |
| T3 child resp. | 3.94 | .86 | 4.85 | 1.01 | 3.78(64) | **<.001** | 3.98 | .77 | 4.92 | 1.05 | 4.07(65) | | **<.001** | 4.25 | .76 | 4.53 | | 1.07 | | .72(65) | .47 | 4.25 | | | | 1.50 | 3.95 | | | .72 | .68(35) | .50 |
| T3 child inv. | 3.79 | .92 | 4.64 | 1.03 | 3.41(64) | **001** | 3.87 | .81 | 4.66 | 1.11 | 3.28(65) | | **.002** | 4.19 | .88 | 4.32 | | 1.09 | | .34(65) | .74 | 3.88 | | | | 1.65 | 3.88 | | | .77 | .01(35) | .99 |
|  | Harm from drugs | | | |  | | Intravenous use | | | |  | | | Replacement therapy | | | | | | | | | Change in substance use during pregnancy | | | | | | | | | |
|  | Yes |  | No |  |  |  | Yes |  | No |  |  | | | Yes | | No | | | | | | Diminished | | | | | Stopped | | | |  | |
|  | % | n | % | n | χ^2^(df) | p | % | n | % | n | χ^2^(df)) | p | | % | n | % | n | | χ^2^(df) | | p | % | | n | | | % | n | | | Fisher’s exact test | |
| Subst. relapses |  |  |  |  | 3.95(1) | **.047** |  |  |  |  | 2.43(1) | .12 | |  |  |  | | | .02(1) | | .88 |  | |  | | |  | |  | | .17 | |
| Yes | 26.1 | 6 | 7.7 | 3 |  |  | 22.2 | 6 | 8.3 | 3 |  |  | | 12.5 | 1 | 14.5 | 8 | |  | |  | 66.7 | | 2 | | | 23.3 | | | 7 |  |  |
| No | 73.8 | 17 | 92.3 | 36 |  |  | 77.8 | 21 | 91.7 | 33 |  |  | | 87.5 | 7 | 85.5 | 47 | |  | |  | 33.3 | | 1 | | | 76.7 | | | 23 |  |  |

Note. Analyses were conducted only for the substance using mothers (PGT and PSS group) ^*^Measured only in the PGT group. AUDIT refers to the measure of alcohol use. R=Pearson’s correlation coefficient. df=degrees of freedom. p-values significant on the level of *p* <. 05 are bolded.
